# An improved SPWM control approach with aid of ant lion optimization for minimizing the THD in multilevel inverters

**DOI:** 10.1038/s41598-024-84678-5

**Published:** 2025-01-15

**Authors:** Alaa M. Abdel-hamed, Abdelrahman M. Nasser, Hamdy Shatla, Amr Refky

**Affiliations:** 1 Electrical Power and Machines Department, Higher Institute of Engineering, El-Shorouk Academy, Cairo, Egypt; 2 Department of Electrical Engineering, Faculty of Engineering, Al-Azhar University, Cairo, Egypt

**Keywords:** Multilevel inverter (MLI), Renewable energy resources (RERs), Sinusoidal pulse width modulation (SPWM), Ant lion optimizer (ALO), THD, GA, PSO, Engineering, Electrical and electronic engineering

## Abstract

This article presents an innovative asymmetric multilevel inverter (MLI) topology that outperforms conventional counterparts. The introduced topology presents a breakthrough in implementing power electronics control by maximizing specific levels while minimizing switching components. A cutting-edge control scheme for optimal operation of the cascaded half-bridge MLI is presented. The ant lion optimization (ALO) algorithm was implemented to optimize the switching control to reduce the total harmonic distortion (THD) and improve power quality. For verification, the performance and effectiveness of the ALO technique are assessed by comparing its results to those obtained using the simplified sinusoidal pulse width modulation (SSPWM) technique, genetic algorithm (GA), and particle swarm optimization (PSO) in existing literature. Simulation results verified the efficacy of ALO in finding the optimal parameters. The suggested method showcases a remarkable reduction in the THD compared to SSPWM. The quality of the resulting waveform was enhanced, and both filter size and cost were significantly reduced. To meet stringent IEEE standards, an LC filter has been designed with minimal size and proper requirements. Experimental results validation of the suggested scheme, using a dSPACE R&D controller board unequivocally, confirmed its robustness and effectiveness. This groundbreaking study not only introduces a superior asymmetric MLI topology but also validates its exceptional performance through comprehensive analysis and experimentation. The experimental waveforms showed good matching with the simulation outcomes. The findings hold immense promise for advancing the field of power system control and revolutionizing the designing and implementation of efficient and cost-effective inverter systems.

## Introduction

The substantial growth of the global population has intensified the utilization of hydrocarbon-based fuels which not only contributes to the phenomenon of global warming and the emission of CO_2_, thereby exerting a detrimental impact on the environment, but has also been demonstrated to represent a vulnerable aspect of economic growth and the industrial revolution^1^. The provision of a consistent and readily available supply of electrical energy constitutes a fundamental pillar for the sustainability of civilization in the long term, encompassing its financial aspects^2^. Owing to the proliferation of users and the emergence of high-power sectors, the demand for electrical energy has experienced a substantial increase throughout recent decades. This conventional means of energy generation has engendered a significant upsurge in environmental pollution^3^. Consequently, the integration of small-scale and large-scale green energy resources into the electricity system has witnessed a remarkable escalation. The global community has observed a transition within the power system, whereby conventional energy resources such as furnace oil and coal have been supplanted by Renewable Energy Resources (RERs), also referred to as non-conventional energy resources, over the past few decades^4^. As a result of the sudden augmentation of RERs and the industrial-sector shift towards non-conventional resources like solar, wind, micro-hydro, biogas, and other RERs^5^, research endeavors have increasingly focused on enhancing power electronic converters. This approach aims to enable network operators to uphold the reliability and stability of the electrical power system within predetermined limitations^6^.

Multi-Level Inverters (MLIs) have a huge effect on several industrial applications, such as the control of electric motors, and power system applications supplied by RERs, especially solar energy^7,8^. MLIs are strongly recommended to cover the disadvantages of conventional inverters, such as the large switching losses and low efficiency^9,10^. The idea of an MLI is to use a modulation technique for producing a staircase AC waveform from a DC source with low THD and low stress on switches^11,12^. MLI has various benefits as compared to the conventional two-level inverters, such as higher efficiency, lower THD in the resulting output voltage, less rate of change of voltage for time in the resulting output voltage, reduction in filter requirements, and lower electromagnetic interference problems^13,14^. Their benefits also include the possibility of avoiding using the step-up transformer in high-voltage grid-connected applications^15–17^. RERs like solar cells and fuel cells can be easily integrated with MLIs; besides, the common mode voltage is lower at the application neutral^18,19^.

The cascaded H-bridge MLI, neutral point MLI, flying capacitor MLI, and generalized MLI are the most popular conventional MLIs^20–22^. These MLIs have various drawbacks, such as implementing many switches that make the gate drive complex, using several capacitors, and the existence of clamping diodes. This could make the inverter bulky, and the system would become more expensive and complicated^23,24^.

Recently, new topologies have been used to overcome the drawbacks of conventional MLIs^25,26^ The most recent MLI topologies have two tracks, the first is to reduce the switching components as much as possible regardless of the components’ rating, and the second is to reduce the components’ rating regardless their number^18,27,28^.

Umashankar et al.^29^, introduced a new 7-level MLI topology. This topology ensured reduced size, minimum switching losses, and accordingly reduced cost of installation by implementing the least number of trigger circuits of the gate and unidirectional switches. The topology is applied for various applications, such as RE and drives. The carrier-based PWM method is used to compare and examine the performance of this topology with the performance of the other 7-level topologies of reduced switching that exist in the literature and the conventional cascaded MLI^30^. The designed topology is simple, has very few components, and has a lower THD than asymmetric and conventional symmetric topologies. A MATLAB/SIMULINK software was implemented to validate the results.

Hanyang Yu et al.^31^, presented a hybrid 7-level converter that is suitable for high power ratings and low voltage applications. It is based on the T-type converter and H-bridge cascade. Its operation and conduction paths were investigated. The mathematical formulation for the conditions of the voltage balance is realized based on SPWM. Results indicated that there is a balance in floating capacitor voltages when the modulation factor (${m}_{i}$) is lower than 0.82 for implementing SPWM. For regulating floating-capacitor voltages, Space Vector Modulation (SVM) is used to gain the merits of redundant switching vectors. In this method, there are no further requirements for controlling the capacitor voltage. The improvement in DC voltage utilization was analyzed in this topology. Results verified the feasibility of implementing SVM and SPWM methodologies for this converter.

Khudair et al.^32^, suggested a new MLI to reduce the total number of switches to alleviate the challenges of the huge size, the significant increase in losses, and the high cost. A cascaded MLI implementing lower devices is described. The suggested MLI showed improved performance with minimized THD. The introduced approach was validated via the MATLAB/Simulink software.

Quayyoom et al.^33^, introduced a new 7-level topology of MLI. Seven levels and boosting voltages up to 1.5 times when applying a DC source can be produced using this topology. Two self-balanced types of switched capacitors and two capacitors of DC link types with neutral points were applied with nine switches. Level-Shifted Modulation (LSM) and Modified Nearest Level (MNL) control methods were applied to control the output voltage. The operation of the suggested topology and the capacitor design are explained and analyzed in detail. The leakage current was eliminated, particularly in solar PV applications, while the applied voltage stress on switches was reduced in this topology. This topology is compared with other ones in the literature in terms of the applied voltage stress on switches and the component numbers for the voltage gain. For performance verification, simulation results are introduced and compared in terms of the THD and efficiency. This introduced MLI topology is better in the gate drive circuitry, DC sources, number of semiconductor switches, THD, efficiency, and other aspects.

Andres et al.^34^, presented a control method for a three-phase cascaded H-bridge inverter. This method compensates for the reactive power and reduces the harmonic currents that are caused by non-linear loads. A generalized model and controller for a three-phase cascaded H-bridge inverter were designed. The designed model aims to comprise a current tracking loop, in addition to regulation and balance voltage loops for all H-bridges. The current reference has been created so that, in a steady condition, it becomes proportional to the fundamental component of the line voltage. To ensure the regulation and balance of the capacitor voltages involved, the suggested controller is designed with exterior voltage loops.

Ebrahimi et al.^35^ proposed a novel topology of a cascaded Multilevel Converter (MLC) based on a cascaded connection of the units of a 1-phase sub-MLC and full bridge converters. In comparison with the classical MLC, the number of switches, dc voltage source, converter cost, and installation area is considerably reduced with the increase of voltage steps. Three methods are introduced to determine the magnitudes of the dc voltage source. The configuration of the introduced topology is optimized to use a minimized number of dc voltage sources and switches and yield output voltage steps of high number. The proposed MLC is verified experimentally implementing a 1-phase 49-level converter. The authors in^36^ proposed a new cascaded MLC topology. The topology reduces the number of diodes, switches, IGBTs, and DC voltage sources when the output-voltage level increases. A proposed algorithm to find the dc magnitudes of dc voltage sources is introduced. The suggested topology is tuned for various control objectives to obtain the maximum output-voltage levels. The proposed MLC is verified experimentally implementing a 1-phase 125-level proto-type converter. The topologies in^35^ and^36^ feature each basic unit with two switches and use a single H-bridge for each basic unit.

Rupali Mohanty et al.^37^ proposed an improved Black Window Optimization (BWO) technique to obtain the optimum firing angles of a minimized structure MLI. The firing angles are optimized to reduce the THD of the resulting output voltage waveform of the MLI. The traditional BWO algorithm was modified and implemented to minimize the THD with fewer tuning parameters, fewer equations, and a faster convergence rate than the other nature-inspired algorithms implemented in the literature. The effectiveness of the proposed BWO technique is verified by solving the optimization problem for 15-level MLI using the Genetic Algorithm (GA), Bacterial Foraging Optimization (BFO) technique, and Particle Swarm Optimization (PSO) technique. The results of these algorithms are compared with those generated using the proposed BWO algorithm. The results were verified via a hardware setup in the laboratory.

Therefore, this paper introduces an improved SPWM control scheme to control a half-bridge MLI. The MLI is designed and simulated based on the suggested control scheme. The optimal switching for the Cascaded H-Bridge MLI (CHB-MLI) to minimize the THD is achieved using the ALO algorithm. The GA and PSO are implemented for comparison and to verify the performance and effectiveness of the suggested approach. The suggested approach is compared with the SSPWM technique implemented in the literature. An experimental setup is constructed to verify the results obtained using the suggested scheme.

The CHB-MLI topology was chosen due to its minimal number of electrical switching components. This topology offers a higher number of resulting output voltage levels while requiring fewer switching components compared to conventional MLIs. The suggested approach only requires three signals, making it easy to implement in real-time systems. It relies on a specifically designed MATLAB code, which allows for seamless integration with Artificial Intelligence (AI) techniques to enhance the inverter’s performance and improve the electrical power quality of the output resulting waveform.

The core contribution of the paper can be specified as follows:

1. A model of reduced THD for a three-phase seven-level cascaded half-bridge MLI with reduced power switching components.
2. Design the suggested SPWM control scheme of MLI.
3. Comparison of the suggested SPWM approach and the SSPWM technique mentioned in the literature.
4. Implementation of the ALO technique to optimize the switching of the MLI, thereby reducing THD and improving power quality.
5. Comparative analysis of the ALO results with those produced using the GA and PSO to assess the performance and effectiveness of the optimization technique.
6. Development of an optimized LC filter design specifically tailored to minimize harmonics in the seven-level MLI inverter.
7. Performance evaluation and experimental validation for the suggested model.

The remainder sections of this article are organized as follows: The model of the suggested MIL is provided in the “System modeling” section. The methodology of the SSPWM technique is presented in the “Methodology of the SSPWM technique” section. “Methodology of the suggested SPWM approach” section introduces the methodology of the suggested SPWM technique. Modeling the implemented ALO algorithm is provided in the “ALO algorithm modeling” section. “Harmonics mitigation techniques” section introduces the method of reducing harmonics and how to implement it with the adopted MLI. Simulation results, discussion, and comparison of the suggested approach with the SSPWM technique are provided in the “Simulation results and discussion” section. The experimental validation results are provided in the “Experimental validation” section. Finally, the conclusion of this paper is provided in the “Conclusion” section.

### System modelling

**Fig. 1:**
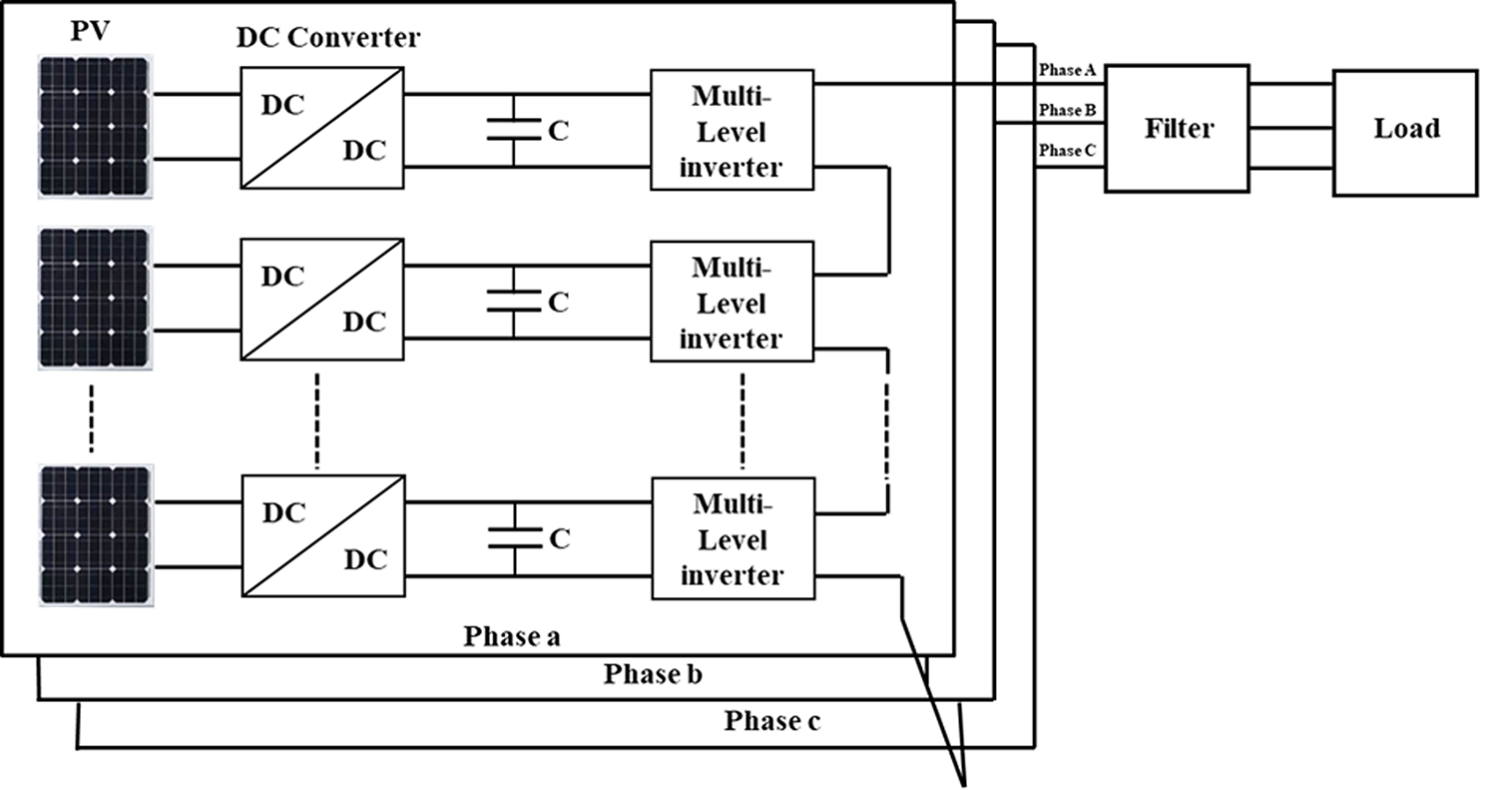
Schematic representation of a PV system integrated with a standalone load.

With the increase in using RERs such as PV, the controlling of MLI becomes more complex. So, an accurate and fast control method is needed to effectively control the inverter. Figure 1 demonstrates the schematic representation of a PV system integrated with a standalone load. In a DC-link bus, the voltage of traditional two-level and three-level inverter systems is 1.5 times the normal output voltage^38^. Large LC filter circuits are needed at the inverter end of two-level and three-level inverters to get sinusoidal output voltage from them. Over the past two decades, MLI topologies have overcome the drawbacks of two- and three-level inverter topologies^39^.

### Modulation techniques

**Fig. 2:**
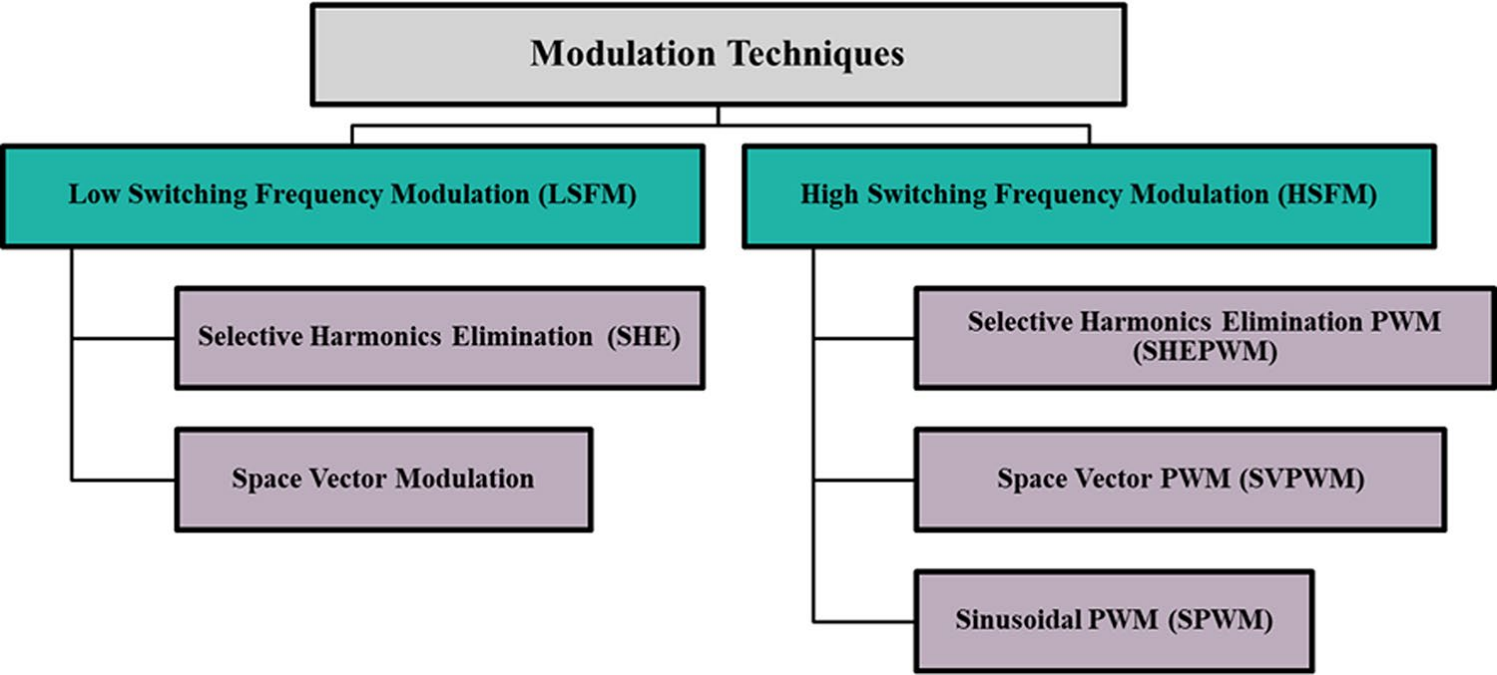
Modulation techniques for controlling the MLIs.

Modulation techniques are used to control MLIs, which can be classified according to switching frequency, as shown in Fig. 2. LSFM is suitable for high-power applications^40^. To eliminate the selective low-order harmonics for certain levels, the number of nonlinear equations can be solved by using AI techniques. Analytical methods such as Newton Raphson and the resultant method can also be used for eliminating the selective harmonics^41,42^.

HSFM is suitable for low-power applications. It shifts the low-order harmonics and reduces the THD by implementing a filter with a small size^43^. These control techniques are commonly used in a variety of MLI configurations, but they cannot be directly applicable to the CHB-MLI investigated in this paper. The adopted CHB-MLI requires the same number of carrier signals as the total number of levels, minus one. To address this problem, the paper suggests a different approach that simplifies the modulation control scheme. This method reduces the required number of carrier signals to one, regardless of the inverter’s levels. By dramatically reducing the total number of carriers, this method becomes much more manageable for use in practical applications.

### Operation of the three-phase CHB-MLI topology

**Fig. 3:**
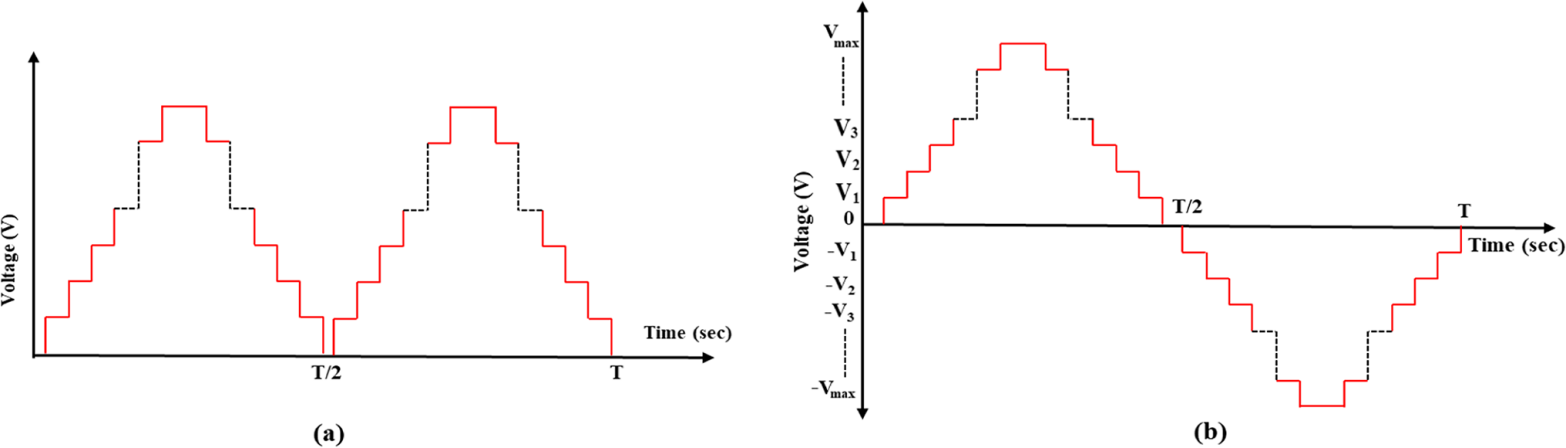
(**a**) Output voltage of level generation-stage (**b**) Inverter AC voltage after H-bridge.

The presented MLI consists of two stages. The first stage is the main stage, and the second is the polarity stage. This type can produce a higher total number of voltage levels with a lower number of switches but a higher rating. The output voltage in the inverter can be generated after the polarity stage as Fig. 3 a indicates^44^. The main stage consists of several core units; each unit consists of only two switches in series. Each unit can produce only two levels of values [0 or ${V}_{dc}$] and has a separate DC voltage source. The voltage produced by each unit will be added so that the total voltage after the main stage will be zero or positive. The polarity stage is a conventional H-bridge that is implemented to produce the voltage from the main stage as the same in the first half cycle and reverse it in the second half cycle to make the DC voltage as an AC voltage as Fig. 3 b indicates. Table 1 indicates the possible switching values of the main stage and the auxiliary stage to produce the desired voltage.

**Table 1:** Switching status of the level generation stage and the polarity stage.

V_out_		Level generation stage						Polarity stage			
		S_1_	S_2_	S_3_	S_4_	S_2n-1_	S_2n_	Q_1_	Q_2_	Q_3_	Q_4_
0	0	0	1	0	1	0	1	1	1	0	0
V_1_	V_dc_	1	0	0	1	0	1	1	1	0	0
V_2_	2V_dc_	0	1	1	0	0	1	1	1	0	0
V_1_ + V_2_	3V_dc_	1	0	1	0	0	1	1	1	0	0
V_3_	4V_dc_	0	1	0	1	1	0	1	1	0	0
V1 + V_3_	5V_dc_	1	0	0	1	1	0	1	1	0	0
V_2_ + V_3_	6V_dc_	0	1	1	0	1	0	1	1	0	0
V_1_ + V_2_ + V_3_	7V_dc_	1	0	1	0	1	0	1	1	0	0

The total voltage ${V}_{odc}$ can be obtained from the level generation stage according to Eqs. (1)$-$(3), which give the total resulting voltage.

1 $$V_{odc} = \mathop \sum \limits_{i = 1}^{n} v_{oi}$$

2 $$m = 2N_{step} - 1{ };{ }N_{cell} = n{ }$$

3 $$S = 2n + 4$$

where ${N}_{cell}$, $n$,${N}_{step}$,$S$ represent the number of core cells, DC voltage sources, switches, and steps, respectively, ${V}_{dc}$ is the value of DC sources, ${v}_{{o}_{1}}$ is the resulting voltage of the core unit, and ${v}_{{o}_{\text{n}}}$ is the resulting voltage for the $n$ unit.

There are many techniques implemented for supplying the MLI. The inverter is feed by equal DC sources with a magnitude of (${V}_{dc}$). If the inverter consists of (*n*) units, the total number of levels (*m*) can be obtained according to Eq. (4).

4 $$N_{step} = n + 1;\;\;{ }m = 2n + 1$$

The percentage reduction ($R\%$) in the total number of switches for each phase of the inverter is calculated using Eq. (5).

5 $$R{\text{ \% }} = \left[ {1 - \frac{m + 3}{{2\left( {m - 1} \right)}}} \right]{*}100$$

The maximum voltage can be calculated from Eq. (6).

6 $$V_{o,max} = n.V_{dc}$$

If DC sources are different in magnitude, then the total number of levels can be increased without adding more units. The DC sources can be chosen according to an arithmetical sequence with a factor of 2 or 3 given by Eqs. (7) and (8).

7 $$N_{step} = 2^{n} ,\;{ }m = 2^{n + 1} - 1,\;V_{j} = \left( {2^{j - 1} } \right).V_{dc} \;\;for{ }\;j = 1,2,3,.....n$$

8 $$N_{step} = \frac{{3^{n} - 1}}{2},\;{ }m = 3^{n} ,\;V_{j} = \left( {3^{j - 1} } \right).V_{dc} \;\;for\;{ }j = 1,2,3,.....n$$

The reduction in the total number of switching devices in each phase is calculated according to Eqs. (9) and (10).

9 $$R{\text{\% }} = \left[ {1 - \frac{{ln\left( {2m + 2} \right)}}{{ln\left( 2 \right).\left( {m - 1} \right)}}} \right]{*}100$$

10 $$R{\text{\% }} = \left[ {1 - \frac{{{\text{ln}}\left( {m - 3} \right)^{2} + 4}}{{2\left( {m - 1} \right)}}} \right]{*}100$$

The maximum generated voltage can be calculated from Eqs. (11) and (12).

11 $$V_{o,max} = (2^{n - 1} ).V_{dc}$$

12 $$V_{o,max} = \left( {\frac{{3^{n} - 1}}{2}} \right).V_{dc}$$

For unequal DC sources, the total number of switches can be calculated using Eq. (13)

13 $$N_{step} = 2n,\;m = 4n - 1,\;V_{j} = j.V_{dc} \;\;for\;{ }j = 1,2,3,.....n$$

The percentage reduction ($R{\text{\% }}$) in the number of switching devices per phase of the inverter can be calculated using Eq. (14).

14 $$R{\text{\% }} = \left[ {1 - \frac{{\left( {{\text{m}} + 9} \right)}}{{4\left( {m - 1} \right)}}} \right]{*}100$$

The maximum voltage generated can be calculated using Eq. (15).

15 $$V_{o,max} = 2\left( {n - 1} \right).V_{dc}$$

The peak inverse voltage for each switch $PIV_{n}$ is indicated by Eq. (16).

16 $$PIV_{j} = V_{{dc_{i} }} \;\;for\;{ }j = 1,2,3....N_{cell} { },\;{ }i = 1,2,3......n.$$

### Methodology of the SSPWM technique

Reference^45^ suggested a strategy for simplifying the switching generation process of the inverter. In contrast to the SPWM which requires ($m-1$) carrier waves, only a single-carrier signal is compared with the modulating signal, so it can be called SSPWM. The modulating signal is a rectified sine wave, which has a peak amplitude and frequency of ${A}_{m}$ and ${f}_{m}$ respectively. The carrier signal is a triangle wave, which has a peak amplitude and frequency of (${A}_{c}$ = 1) and ${f}_{c}$ respectively. The amplitude modulation factor (${m}_{i}$) is the ratio between ${A}_{m}$ and ${A}_{c}$ while the frequency modulation factor (${m}_{fr}$) is the ratio between ${f}_{c}$ and ${f}_{m}$.

**Fig. 4:**
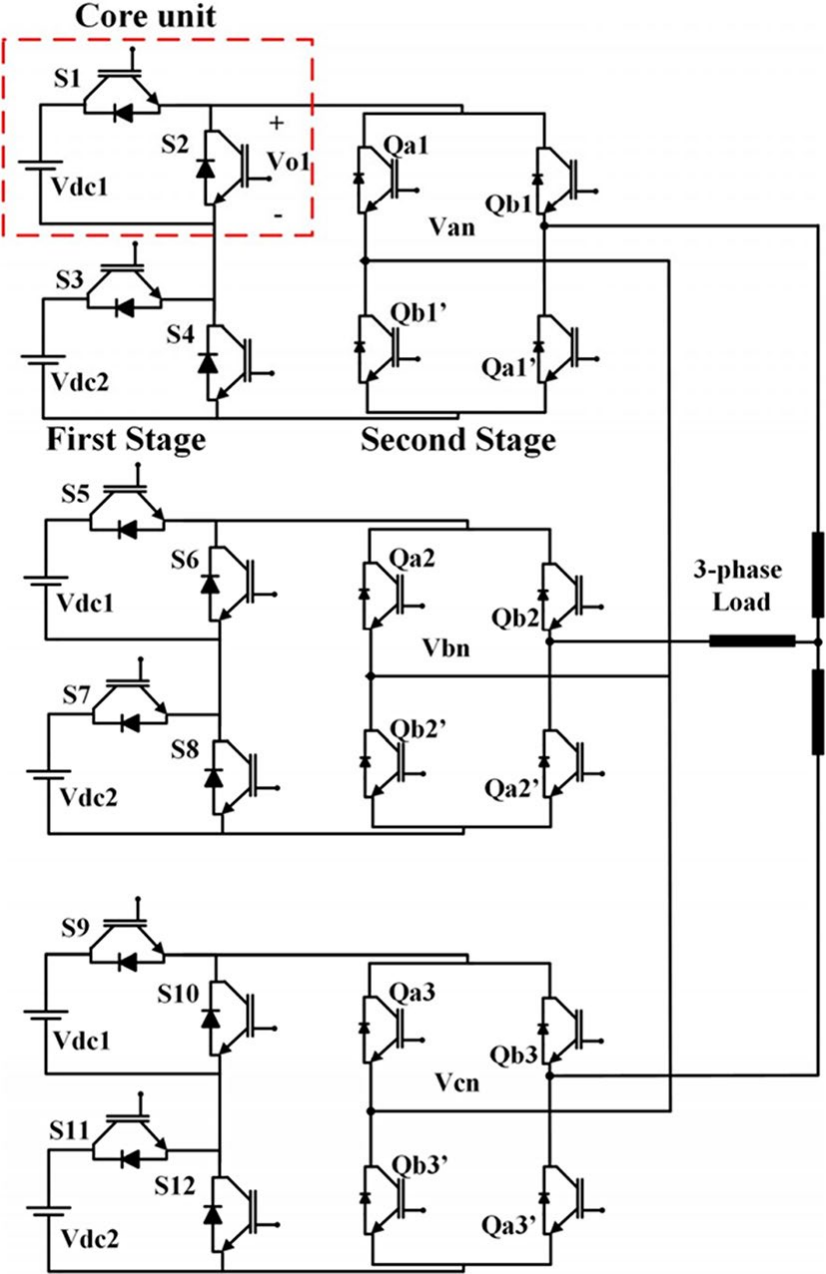
Schematic diagram for a three-phase asymmetric seven-level CHB-MLI.

**Fig. 5:**
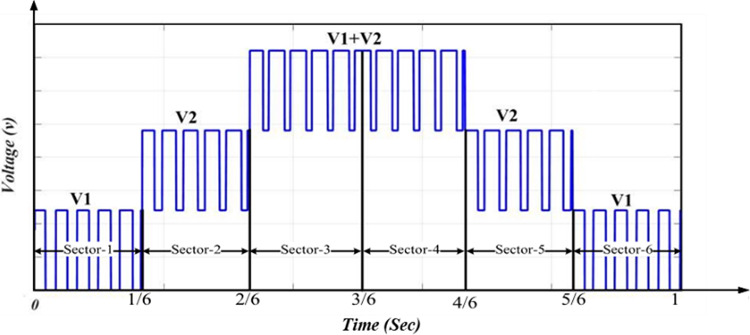
Half-cycle of resulting voltage for the adopted inverter.

**Fig. 6:**
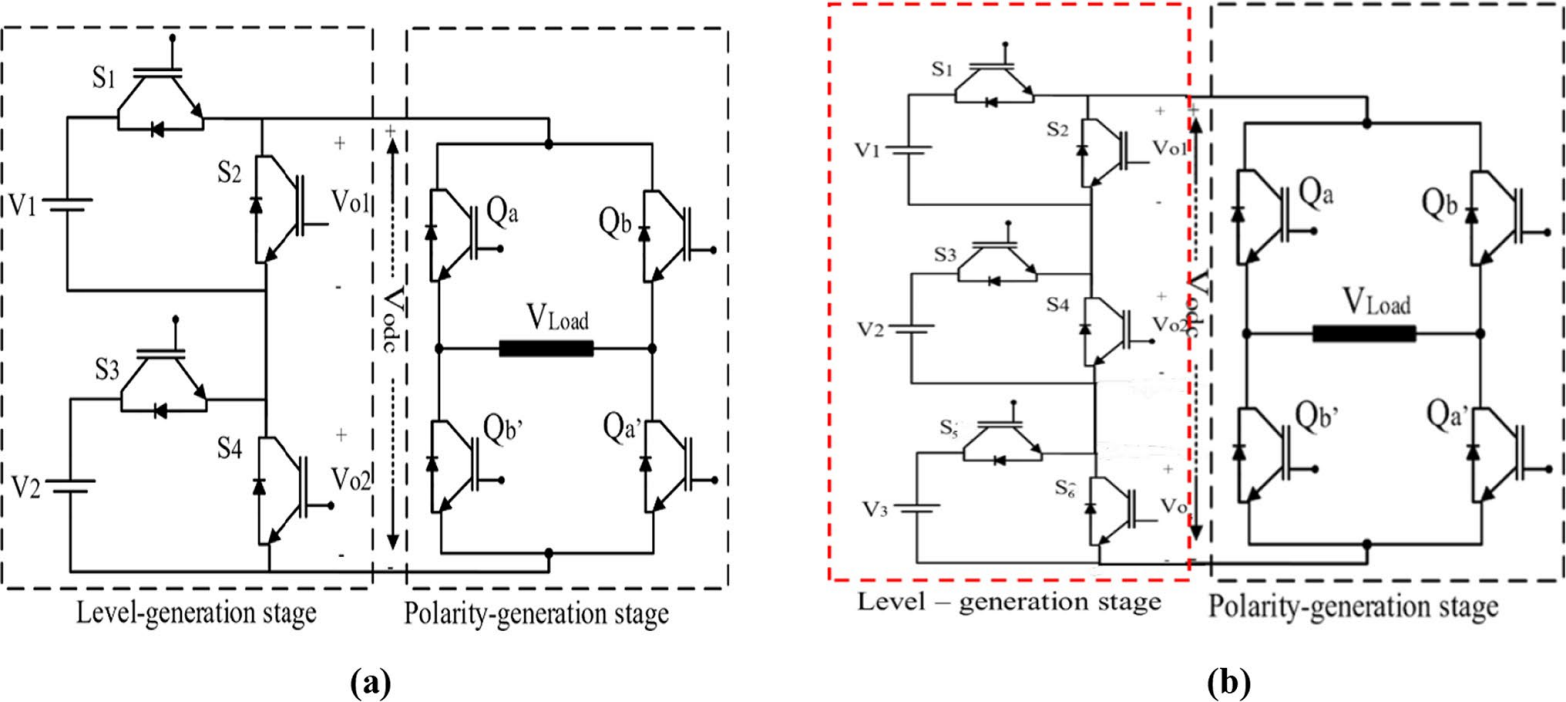
(**a**) Asymmetric 7-level inverter (**b**) Symmetric 7-level inverter.

The schematic diagram of the three-phase MLI is indicated in Fig. 4. The modulation technique can be clarified with a half-cycle of the inverter resulting voltage as shown in Fig. 5, which is divided into 6-sectors with voltage level limits. Figure 6 shows the circuit diagram of the 7-level inverter. It is obvious from Figs. 5 and 6 that sectors 1 and 6 have voltage limits of (${V}_{min}$= 0 and ${V}_{max}=$ ${V}_{1}$) which can be obtained by firing *S*_*1*_ using SPWM pulses. The switching values for the remaining sector are indicated in Table 2.

**Table 2:** Switching table for a 7-level inverter.

V_out_	S_1_	S_3_	Q_a_	Q_b_	Q_a_'	Q_b_'
V_1_	S_PWM_	0	1	0	1	0
V_2_	S_PWM_'	S_PWM_	1	0	1	0
V_1_ + V_2_	S_PWM_	1	1	0	1	0

**Fig. 7:**
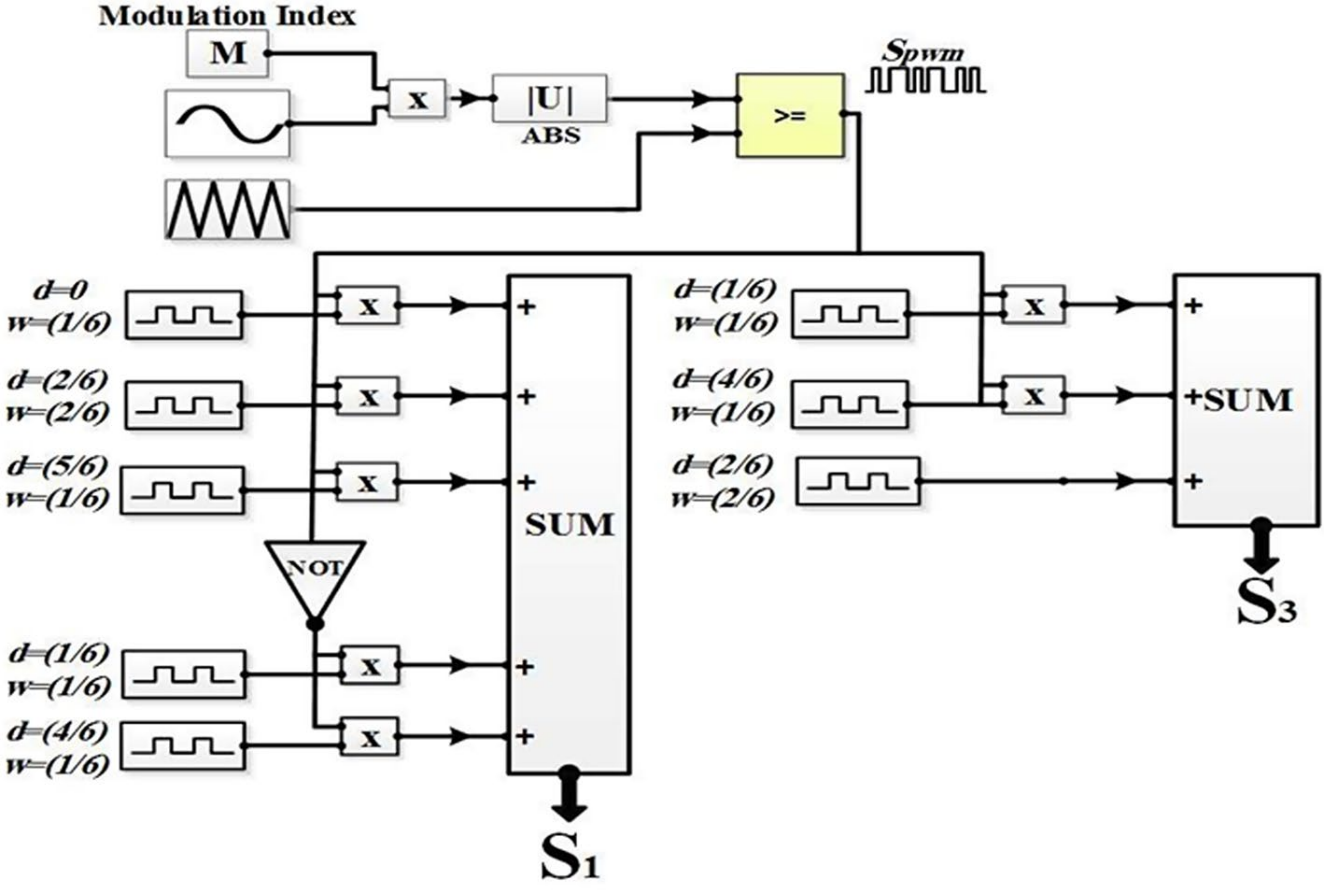
Simulation pattern of SSPWM switching control technique for seven level MLI.

Figure 7 demonstrates the simulation pattern of the switching for the 7-level MLI. Table 2 indicates switching sequence for the seven-level inverter.

For a three-phase system, the output terminals of three single-phase inverters of the half-cascaded type with identical structures can be connected in either delta or star configuration. The voltage between phases A and B V_AB_ can be written as Eq. (17) indicates.

17 $${ }V_{AB} = V_{AN} - V_{BN}$$

18 $$m_{line} = 2m - 1$$

Theoretically, the maximum number of line voltage levels is provided by Eq. (18), where ${m}_{line}$ is the number of line voltage levels. A main advantage of the three-phase systems is the elimination of all triplen harmonic components in the line voltage by the one-third cycle phase shift feature^46^.

### Methodology of the suggested SPWM approach

**Fig. 8:**
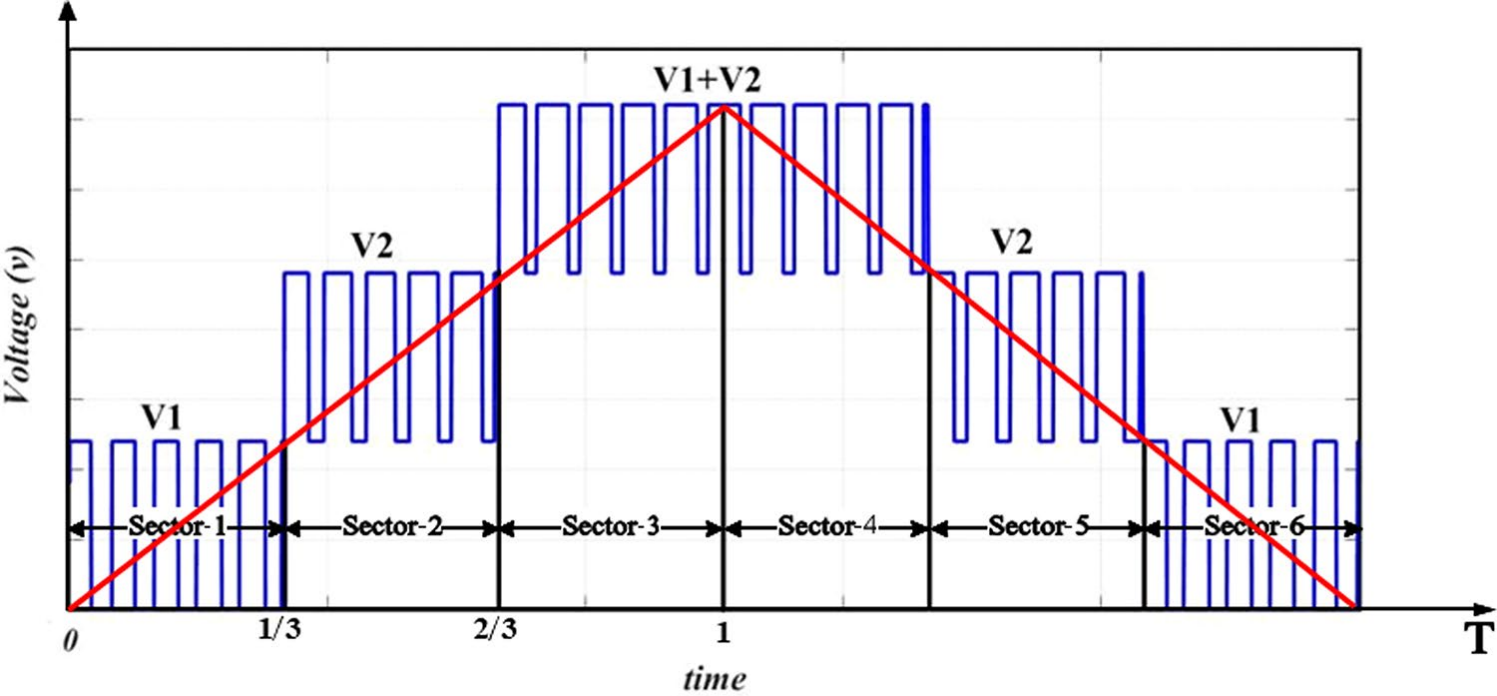
Inverter half-cycle resulting voltage waveform for improved SPWM approach.

**Fig. 9:**
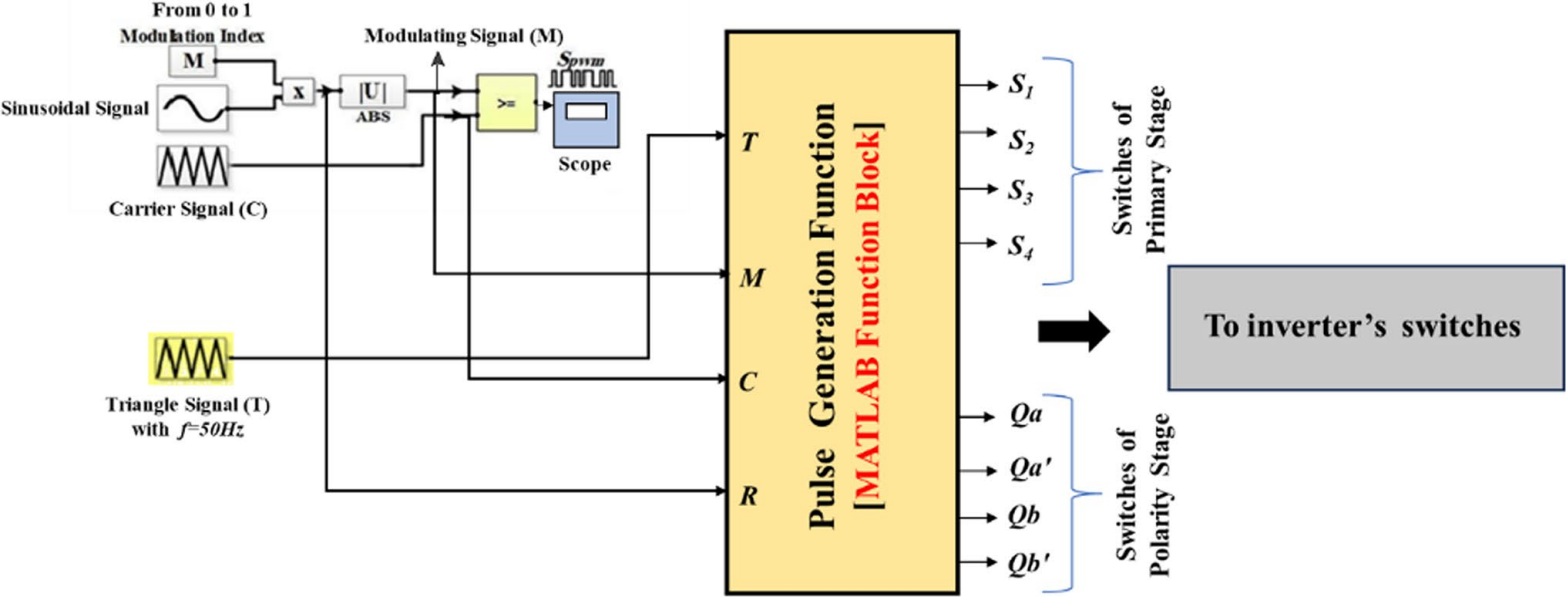
Simulation pattern of suggested SPWM approach for the asymmetric 7-level MLI.

**Fig. 10:**
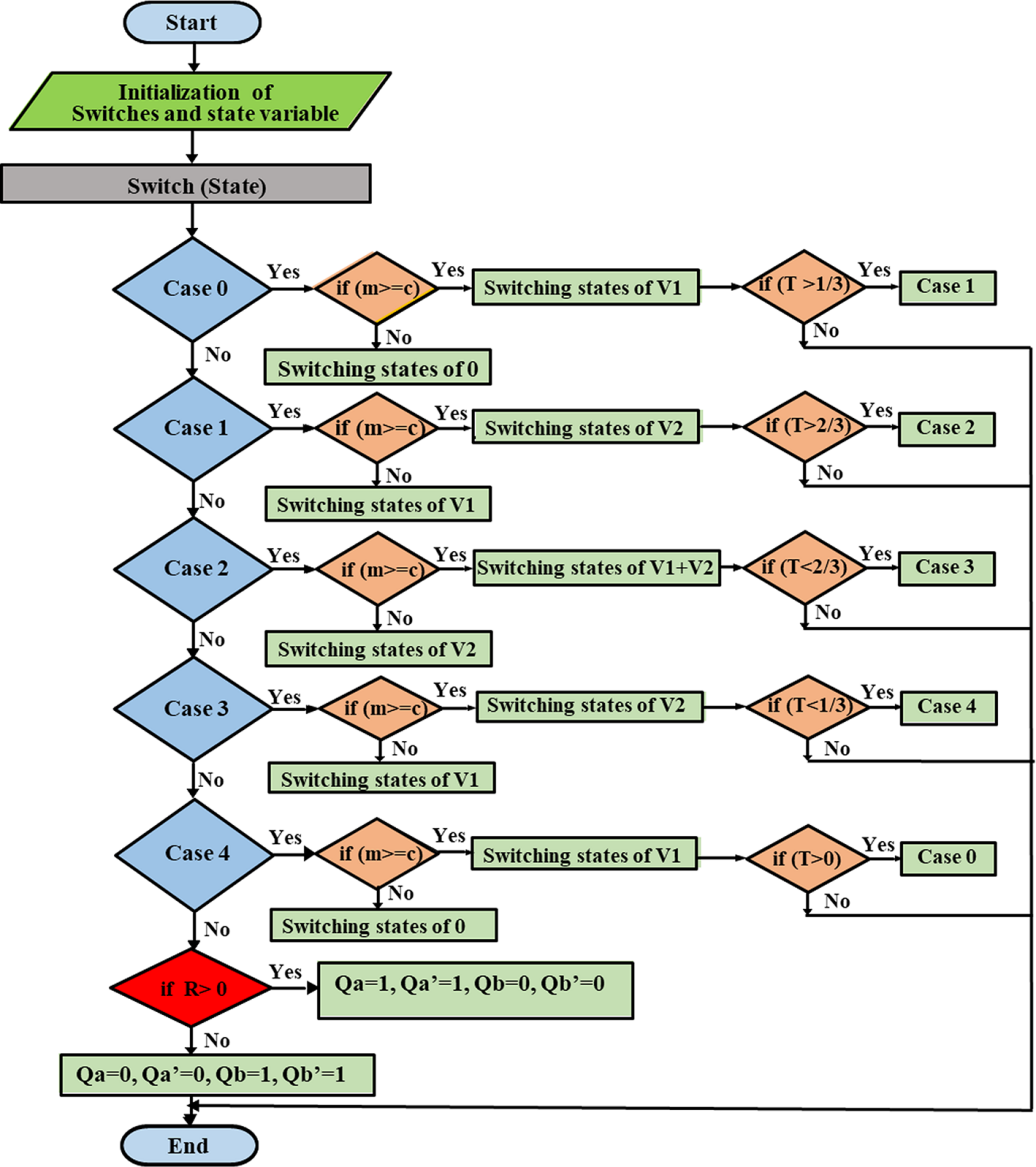
Flow chart of the suggested SPWM approach.

The suggested approach is HSFM which is based on the SPWM. It aims to simplify the pulse generation process of the MLI switches. This requires only one carrier signal to be compared with the modulating signal and a triangle wave with the same frequency of the modulating signal. The slope of the triangle wave is unity, as Fig. 8 indicates. By implementing a MATLAB function block as shown in Fig. 9, the generation process of pulses can be linked with switches via a code that is indicated by the flowchart shown in Fig. 10.

This modification makes the implementation of MLI easier. Besides, it can be experimentally executed more easily and at a low cost. For the three-phase system, the 120° shifting for the three-phase system was carried out using a MATLAB code that was executed in MATLAB function block.

**Fig. 11:**
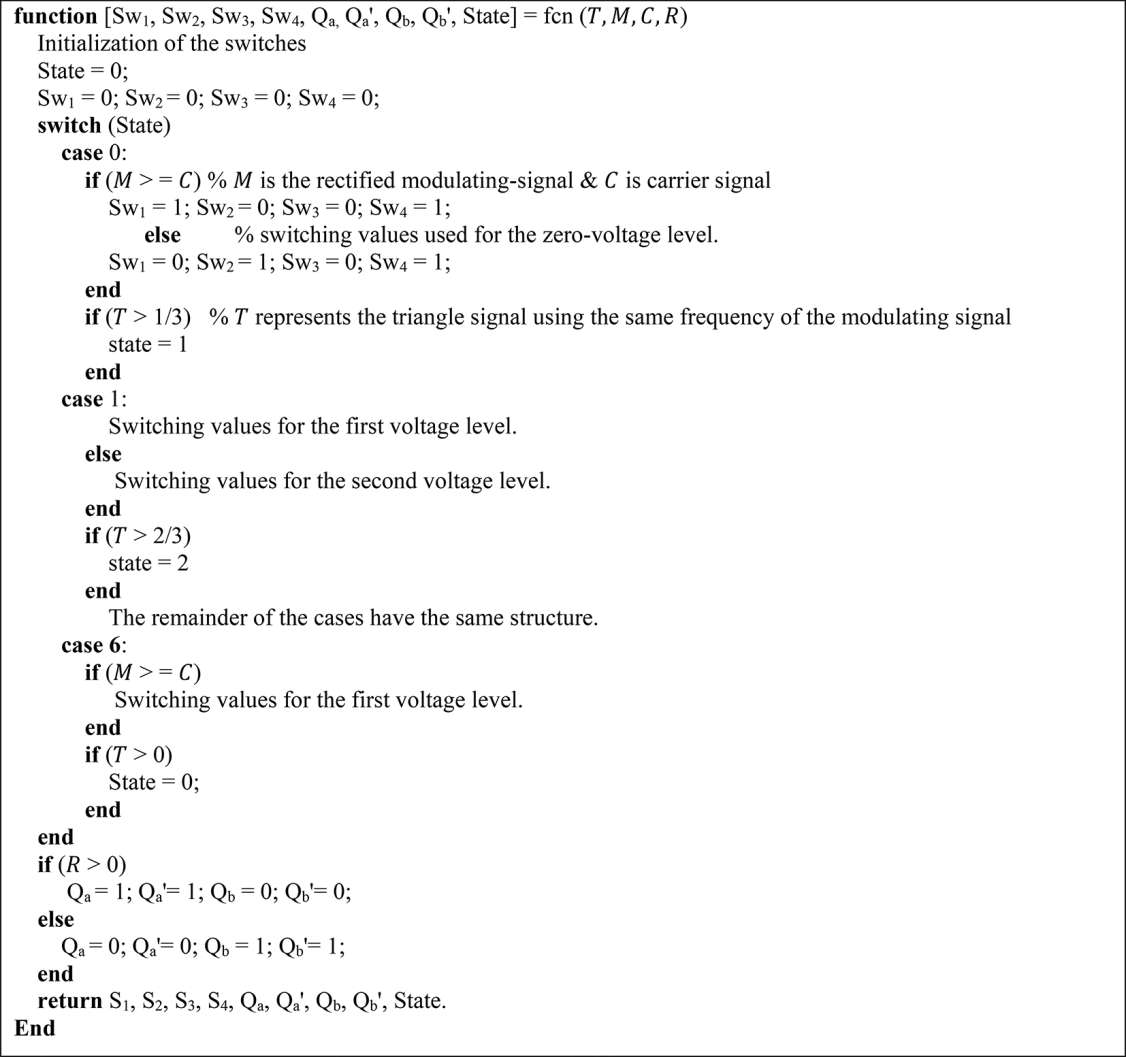
Pseudo-code of the suggested SPWM switching approach.

The pseudo-code presented in Fig. 11 outlines the mathematical model developed to regulate the adopted CHB-MLI. It defines a control approach that governs the switching characteristics of the CHB-MLI based on the input parameters. The pseudocode establishes conditions and switches (*Sw*_*1*_ to *Sw*_*4*_) for each state, which determine the desired configuration of the H-bridge switches in the implemented CHB-MLI. These switches manipulate the desired resulting waveform. Furthermore, the input signals “*T*,” “*M*,” “*C*,” and “*R*” are taken into consideration. These parameters represent system variables or control signals that influence the decision-making process and determine the appropriate switching arrangement for the MLI. The pseudocode also includes an output section that assigns values to the variables (*Sw*_*1*_ to *Sw*_*4*_ and State) based on the current state and the input parameters. These variables are utilized for analysis, monitoring, or controlling the behavior of the MLI.

### ALO algorithm modelling

The ALO algorithm is a nature-inspired optimization technique that mimics the hunting behaviour of ant lions^47^. It is based on the concept of the trap-building mechanism adopted by ant lions to capture their prey. By employing the ALO algorithm, it becomes possible to optimize the control parameters of electric power systems to minimize THD and achieve improved system performance. The methodology of the ALO algorithm involves a population-based search procedure. The algorithm starts by randomly initializing a population of ant lions, each representing a potential solution. These ant lions undergo a series of iterations, simulating their movement and trapping behaviour. During each iteration, if the iteration number is less than the maximum iteration number the lower and upper bounds have been updated using specific Eqs. (19), and (20) ^48^.

19 $$\gamma \left( i \right) = \frac{c\left( i \right)}{R}$$

20 $$\delta \left( i \right) = \frac{d\left( i \right)}{R}$$

where $\gamma (i)$ represents the minimum values of all decision variables at the *i*^th^ iteration; $c(i)$ includes the minimum values of all decision variables at the same iteration; $R$ represents the ratio used to update the lower and upper bounds of the decision variables; $\delta$ represents the maximum value of all decision variables at a specific iteration ($i$); the vector $d(i)$ contains the maximum values of all decision variables for the same iteration ($i$).

The calculation of $\delta$ is problem-specific and not explicitly defined in the provided information. It is likely determined based on the problem’s constraints and the current positions of the ants. The calculation of $d$, like $\delta$, is problem-specific and not explicitly defined in the information provided. The ratio *R* is calculated using Eqs. (21) and (22).

21 $$R = 10^{w} \frac{i}{iter}$$

22 $$w = \left\{ {\begin{array}{*{20}c} {2 i > 0.1*iter} \\ {3 i > 0.5*iter} \\ { 4 i > 0.75*iter} \\ {5 i > 0.9*iter} \\ { 6 i > 0.95*iter} \\ \end{array} } \right.$$

where $w$ is the variable used to calculate the rate at which *R* changes over iterations; $i$ denotes the current iteration number of the ALO algorithm and it is a counter that increments from 1 to the algorithm’s maximum iteration number ($iter$).

Equation (22) in the original description is used to calculate the specific value of $w$, which is determined by the iteration number ($i$). During each iteration, the algorithm updates the antlions’ positions, assesses their fitness, and performs other operations to direct the search to the best solution. The decision variables $\delta$ and $\gamma$ are updated using the following Eqs. (23) and (24).

23 $$\gamma_{m} \left( i \right) = Antlion_{t} \left( i \right) + \gamma \left( i \right)$$

24 $$\delta_{m} \left( i \right) = Antlion_{t} \left( i \right) + \delta \left( i \right)$$

The ant positions are updated using Eq. (25).

25 $$Ant_{n}^{i} = \frac{{R_{l}^{i} + R_{e}^{i} }}{2}$$

In each iteration, the position of the antlion chosen at the *n*^th^ position is denoted as "*n*". Additionally, ${R}_{l}^{i}$ represents a random walk around the antlion selected on the *l*^th^ roulette wheel during the *i*^th^ iteration, while ${R}_{e}^{i}$ represents a random walk around the elite antlion during the same iteration.

The ant lions update their positions based on a combination of local search and global search mechanisms. The local search mechanism allows the ant lions to exploit the local region around their current positions, while the global search mechanism enables them to explore the entire solution space. This combination of exploration and exploitation helps the algorithm converge towards the optimal solution. An antlion is replaced with its corresponding ant, if it becomes fitter, based on Eq. (26).

26 $$Antlion_{l}^{i} = Ant_{m}^{i} {\text{if}} f\left( {Ant_{m}^{i} } \right) > f\left( {Antlion_{l}^{i} } \right)\,$$

**Fig. 12:**
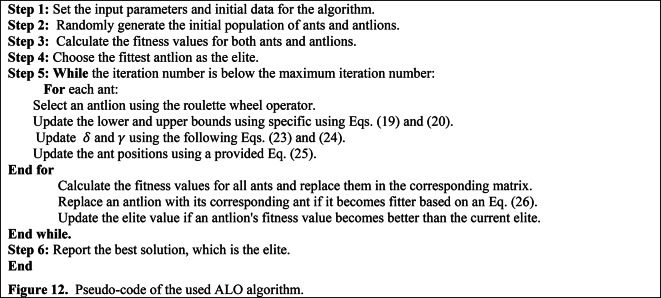
Pseudo-code of the used ALO algorithm.

The elite value is updated if an antlion’s fitness value becomes better than the current elite. The fitness function used in the ALO algorithm is designed to evaluate the quality of each ant lion’s solution. The code provided is an implementation of the ALO algorithm for a specific application. Detailed working procedures are described below as a pseudo-code in Fig. 12.

## Harmonics mitigation techniques

### Optimization techniques

Optimization techniques play a crucial role in reducing harmonics and improving the performance of inverters^49,50^. By employing optimization techniques, it becomes possible to control the operation of the inverter and select the optimal switching patterns, thereby minimizing the generation of harmonics^51^. It is possible to minimize the harmonic content significantly through tuning and selecting the modulation indices and switching patterns. The importance of optimization techniques in reducing harmonics lies in their ability to enhance the efficiency, reliability, and overall performance of inverters. Controlling the inverter operation and selecting the optimal switching patterns can mitigate harmonics leading to reduced losses and improved power quality^52^.

In this paper, the ALO technique will be implemented to optimize the switching control scheme of the adopted MLI. By applying the ALO technique, the control scheme will be fine-tuned to maximize the desired control objectives, such as minimizing harmonics and improving the overall system efficiency. To further ensure the effectiveness and efficiency of the ALO technique, the GA, and PSO technique will be employed.

### Harmonic mitigation filters

**Fig. 13:**
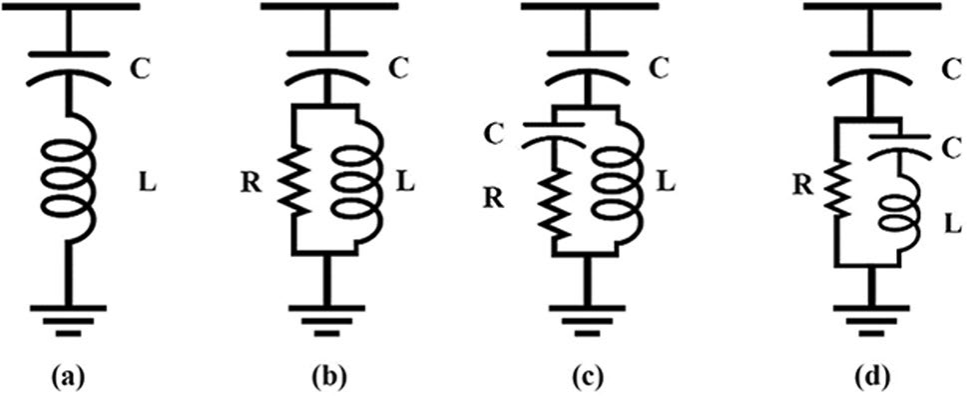
Passive filter types (**a**) single-tuned (**b**) high pass (**c**) double-tuned (**d**) C-type.

Harmonic Filters (HFs) are one of the most practical and efficient methods used for minimizing harmonics^53^. A passive HF is built by combining inductors, capacitors, and/or resistors. They prevent harmonics from flowing through the power system. They are connected as a shunt branch or in series with a load^54^. Passive filters are capacitive at the fundamental frequency for correcting power factors, providing reactive power, and improving the voltage waveform generated by inverters. Figure 13 indicates the four types of passive HFs. These types are the single-tuned, double-tuned, high-pass, and c-type. The most common of them is the single-tuned type filter^55^.

In this paper, an LC filter is used to reduce the THD. The reactive power (${Q}_{cf}$) produced by the capacitor should be less than or equal to 5% of the rated active power (${P}_{r}$)^56^. The maximum size of the filter capacitance can be determined as explained by Eq. (27).

27 $$C_{\max } = \frac{{Q_{cf} }}{{6\pi f_{1.} V_{ph}^{2} }},\;\;Q_{{c_{f} }} \le 5\% P_{r}$$

where ${f}_{1}$, ${V}_{ph}$, and ${P}_{r}$ are the fundamental frequency, rms value of the load phase-voltage, and maximum power demand, respectively.

The filter transfer function (${G}_{f}$) of an LC filter is the ratio of the load phase-current ${i}_{load}$ to the resulting phase voltage ${V}_{i}$ ^55^. The transfer function for a system with an equivalent load resistance (${R}_{load}$) can be expressed as indicated in Eq. (28).

28 $$G_{f} = \frac{{i_{load} \left( s \right)}}{{v_{i} \left( s \right)}} = \frac{1}{{S^{2} \left( {R_{load} L_{f} C_{f} } \right) + S\left( {L_{f} } \right) + R_{load} }}$$

where ${L}_{f}$, and ${C}_{f}$ are the calculated inductance and capacitance values, respectively.

After performing a Fast Fourier Transform (FFT), the dominant individual-voltage harmonics will be identified. This will lead to obtain ${v}_{i}(h)$. For a standalone system, Maximum harmonic current distortion in percent of ${i}_{load}$ will assumed to be 5%, Then the individual current harmonics can then be calculated. Subsequently, the transfer function will be solved to determine ${L}_{fmax}$ by using the previous data. In this case$; s=Jw$.

### Control objective and AI implementation

#### Control objective

The main objective of the optimization technique is to achieve an optimal width for every voltage level. The implemented fitness function to be minimized for obtaining the optimal firing angles is the THD in the resulting output-voltage waveform of the MLI. The mathematical model of the THD is calculated using (29).

29 $$THDv = \frac{{\sqrt {\mathop \sum \nolimits_{h = 3,5,7...}^{\infty } V_{h}^{2} } }}{{V_{o} }}$$

where the symbol ${V}_{h}$ represents the root mean square values of the harmonic voltages, ${V}_{o}$ represents the rms value of the fundamental voltage, and *h* represents the number of harmonics.

The optimality index of the proposed topology is based on the lowest THD values achieved for the 7-level inverter configuration. The efficiency and effectiveness of the implemented control technique were compared against the GA and PSO approaches used in the literature to ensure that significant improvements were attained. By leveraging this advanced control method, the inverter produces the desired AC voltage output using the lowest number of components, while maintaining a simple control scheme. This combined approach allows for a more cost-effective solution compared to alternative MLI designs, making it an attractive option for practical applications.

### Suggested control scheme implementation

**Fig. 14:**
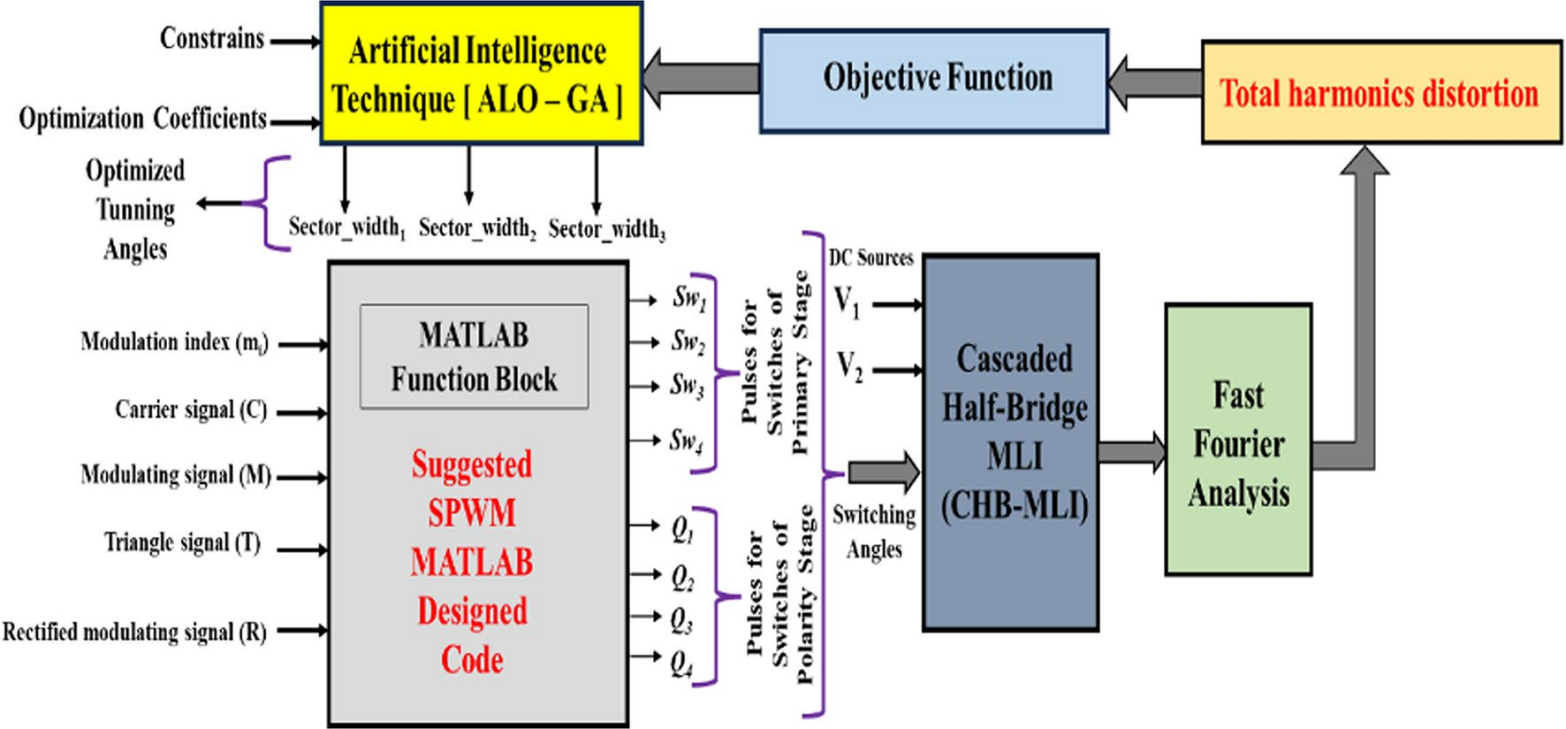
Complete optimal tunning model for the CHB-MLI.

Figure 14 depicts the comprehensive model implemented to find the optimal switching angles for the MLI. These tuned angles play a vital role in generating the required voltage waveforms by appropriately tuning the pulses and DC voltages in the MLI. The pulse generation is extensively explained in the previous Fig. 9. It is implemented using a custom-designed code that utilizes a MATLAB function block. The code relies on three crucial signals: the carrier signal (C), the modulating signal (M), and the triangle signal (T), along with the modulation factor value (*m*_*i*_), to perform the necessary computations and generate the switching angles.

The resulting voltage waveform undergoes Fast Fourier Transform (FFT) analysis, allowing for the calculation of the THD using Eq. (29). Minimizing the THD value is of utmost importance, and this necessitates determining the optimal firing angles through the ALO algorithm, GA and PSO. These techniques utilize the THD values and specific parameters associated with each optimization algorithm. Furthermore, the optimization process is subjected to constraints defined by Eqs. (30) and (31), which impose limits on the values of $Secto{r\_width}_{1}$ to $Secto{r\_width}_{3}$.

30 $$\begin{aligned} & 0 \le Sector\_width_{1} \le 1 \\ & 0 \le Sector\_width_{2} \le 1 \\ \end{aligned}$$

31 $$\begin{aligned} & 0 \le Sector\_width_{3} \le 1 \\ & Sector\_width_{1} < Sector\_width_{2} < Sector\_width_{3} . \\ \end{aligned}$$

To combine the simulation program of the CHB-MLI with the optimization algorithms, the MATLAB environment is employed. The fitness function and the required condition, as stated in Eqs. (29) and (32), need to be satisfied. The condition specifies that the width of all sector values must be arranged in ascending order and lie within the range of 0 and 1.

32 $$0 < Sector\_width_{1} < Sector\_width_{2} < Sector\_width_{3} < 1$$

### ALO implementation

**Fig. 15:**
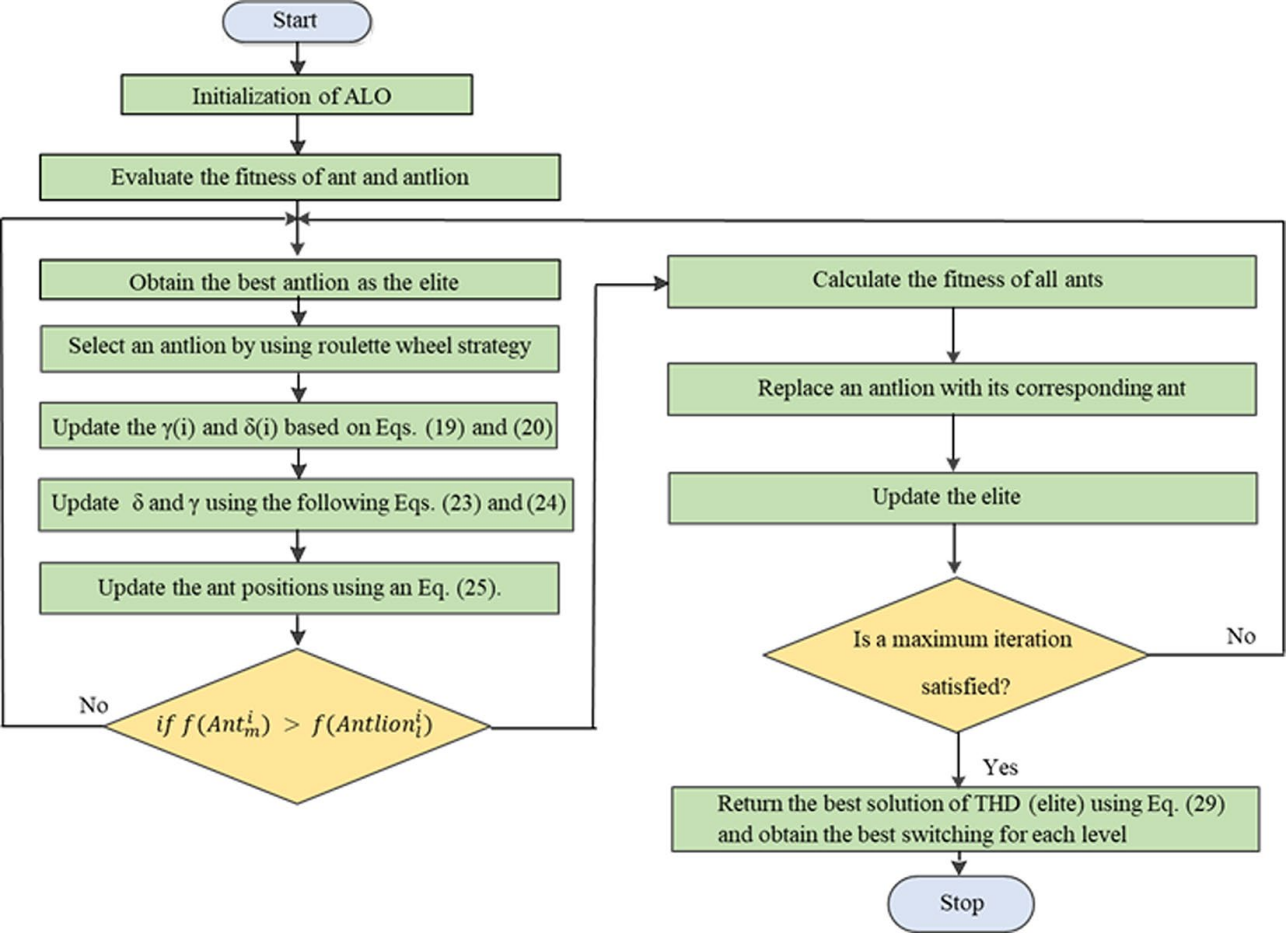
Flow chart of the ALO algorithm.

The objective function presented in Eq. (29) is optimized using the ALO algorithm to control the switching angles of the adopted MLI. The ALO algorithm, as described in the section “ALO algorithm modeling”, is used to find a single solution for each modulation factor value. To cover the entire range of modulation indices, the algorithm is run iteratively multiple times. It then runs 25 independent trials with various settings until the required solutions are close enough together. Figure 12 in the previous section shows the implemented pseudo-code of the ALO technique. This code of the implemented ALO outlines its main stages. Table 3 details the ALO parameters used in the tuning process, which were derived from several experimental trials. Figure 15 demonstrates the flow chart of the implemented ALO algorithm. The ALO was chosen for implementation because of the numerous benefits it provides. These include global optimization capability, simplicity and ease of implementation, fast convergence, robustness, and a balance of exploration and exploitation. Using these advantages, the ALO optimization technique can effectively address the challenges of reducing the THD and improving power quality in the considered system.

**Table 3:** Parameters of the ALO algorithm.

Parameter	Values
Number of ant lions	80
Number of dimensions	3
Number of iterations	250
Mutation probability	0.1

### GA implementation

**Fig. 16:**
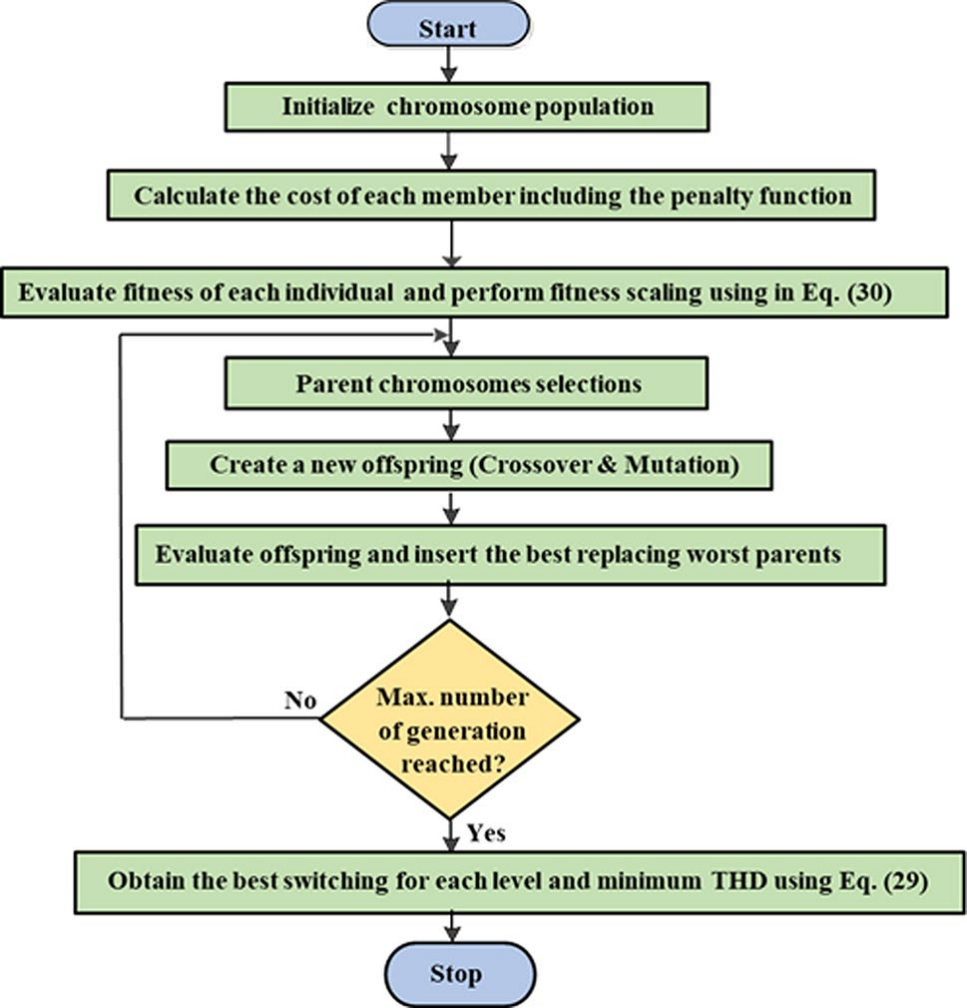
Flowchart of the used GA technique.

The GA technique was used to compare and validate the effectiveness of ALO results. The GA technique described in^57^ is used to determine the optimal firing angles for the improved SPWM control scheme explained in the “Methodology of the suggested SPWM approach” section. The objective function specified in Eq. (29) is used to assess the chromosomes that represent the switching angles. Initially, a random population of chromosomes is generated^58^. The chromosomes are then calculated using the objective function investigated in the sub-section “Control Objective” and indicated by Eq. (29). As discussed in^59^, a new population is chosen based on the objective function by calculating crossover and mutation rates. The evaluation and generation process is repeated automatically until a predetermined termination condition is met, as described in^60^. Through experience and multiple trials, it was determined that mutation should occur with a small probability of 0.1% to 2%, while crossover should occur with a 60% to 90% probability^61^. The GA technique was used in 25-independent trials with various settings until sufficiently close solutions were found. Figure 16 depicts the flow chart of the implemented GA, while Table 4 presents the basic parameters of GA based on various trials.

**Table 4:** Parameters of the GA algorithm.

Parameters	Values
Population size	80
Number of iterations	250
Number of dimensions	3
Number of elite individuals ($nElite$)	50
Cross over rate	0.8
Mutation rate	0.01

### PSO implementation

Particle Swarm Optimization (PSO) is one of the most employed artificial intelligence optimization techniques, drawing inspiration from the foraging behaviour of fish and bird flocks. In this method, a group of particles is randomly generated to create a population. Unlike GA, PSO retains all particles throughout the optimization process. These particles navigate through a multi-dimensional space in search of the optimal solution, each representing a potential answer to the optimization challenge. Their movement is influenced by their velocity^37^. The PSO technique used in^37^ was implemented to compare and validate the effectiveness of the results obtained from the ALO method.

**Fig. 17:**
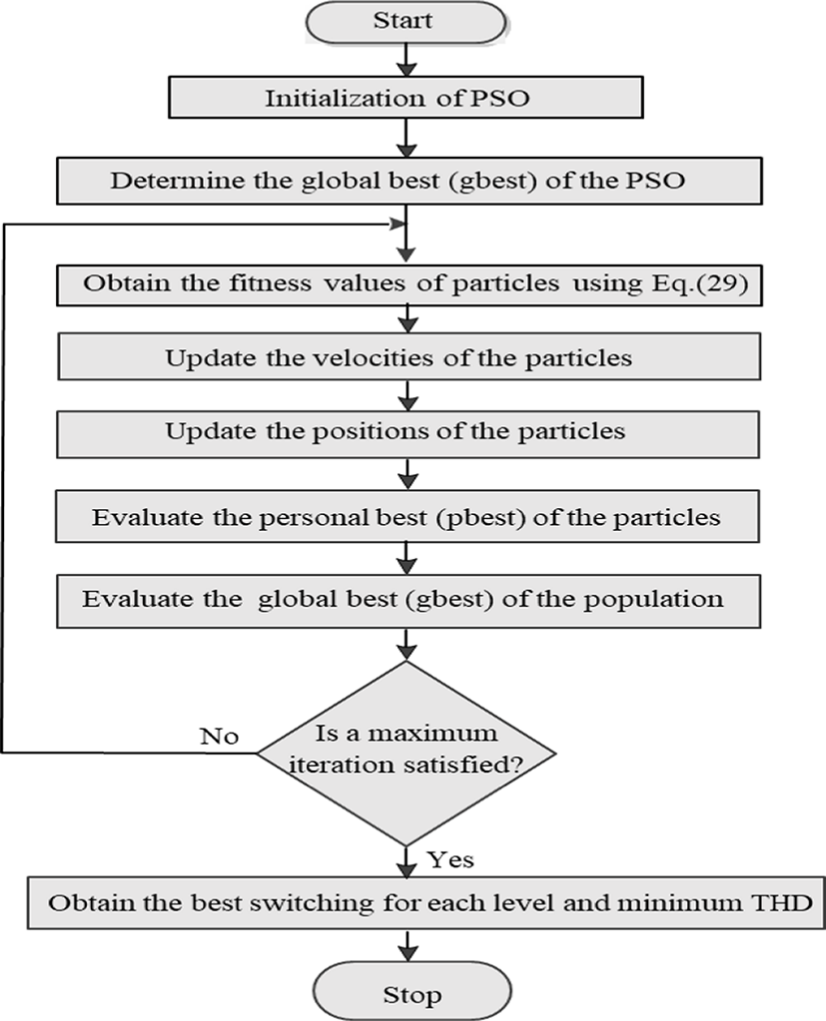
Flowchart of the used PSO technique.

The key phases of the PSO method are illustrated in the flowchart in Fig. 17. The main goal of employing PSO is to determine the optimal width for each voltage level and to minimize the (THD), as indicated in Eq. (29). Results obtained through the PSO algorithm are based on the values presented in Table 5. The PSO technique was used in 25-independent trials with various settings until sufficiently close solutions were found.

**Table 5:** Parameters of the PSO algorithm.

Parameter	Value
Swarm Size	80
Number of dimensions	3
Cognitive Parameter (${C}_{1}$)	2
Social Parameter (${C}_{2}$)	2
Inertia Weight ($w$) [${w}_{min}$, ${w}_{max}$]	[0.2, 0.85]
Maximum Velocity	6
Number of iterations	250
Simulation run time	1.5 s

## Simulation results and discussion

**Fig. 18:**
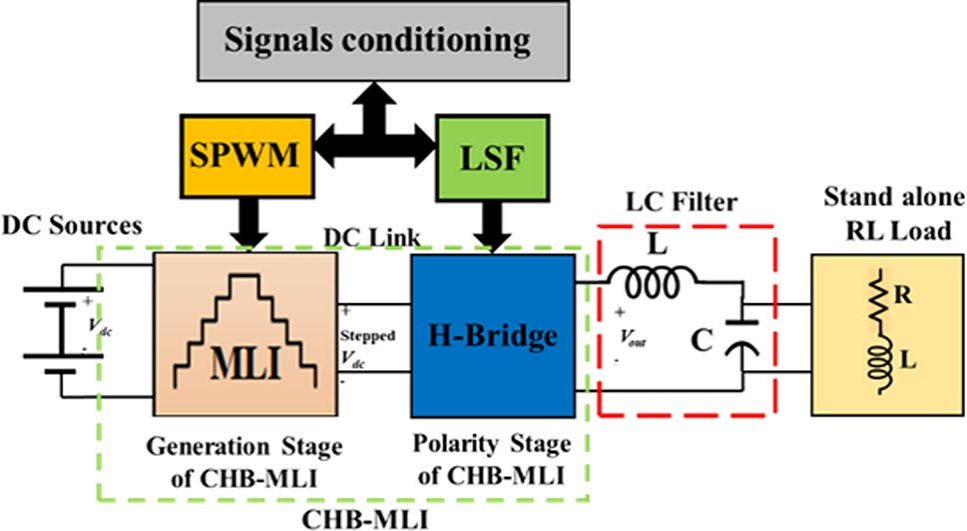
Block diagram for complete system modelling.

The simulation model shown in Fig. 18 is implemented in MATLAB R2017b platform with a laptop with an Intel (R) Core™, i7, CPU at 2.60 GHz, and RAM built with 16 GB. The simulation is performed at a total run-time of 1.5 s. The following subsections describe and explain the simulation results using the SSPWM technique implemented in the literature, the suggested SPWM approach, a comparison of the suggested scheme with the SSPWM technique used in the literature, the optimal firing of switches by implementing the ALO algorithm, and comparison with GA and PSO.

### Results using SSPWM technique implemented in the literature

**Fig. 19:**
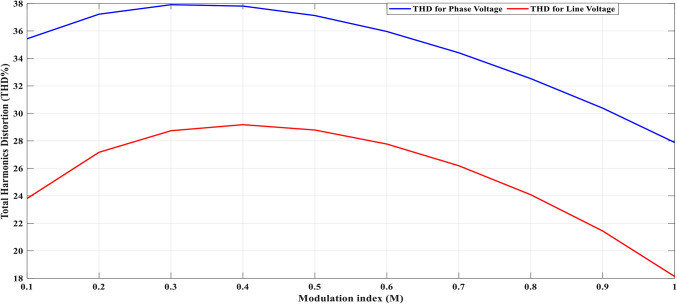
Percent THD versus modulation factor implementing the SSPWM technique.

**Fig. 20:**
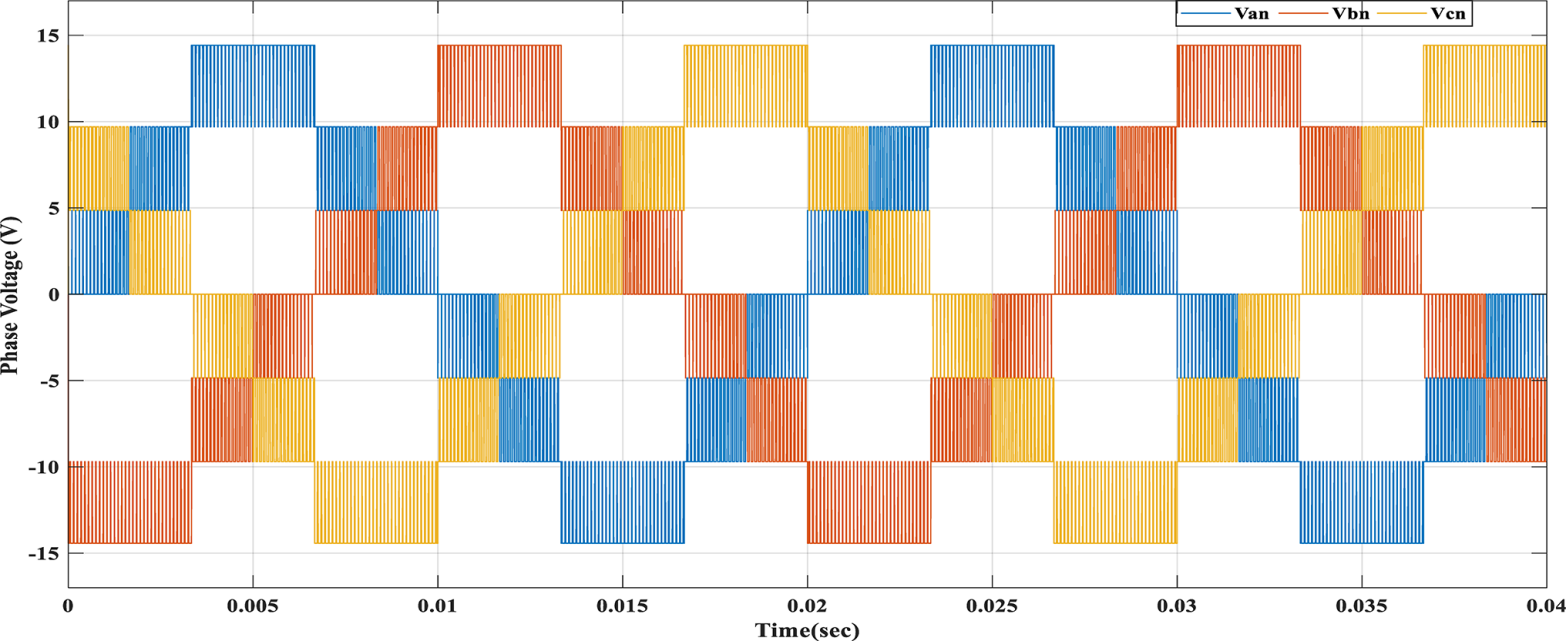
Three phase voltages at ${m}_{i}$= 0.95.

**Fig. 21:**
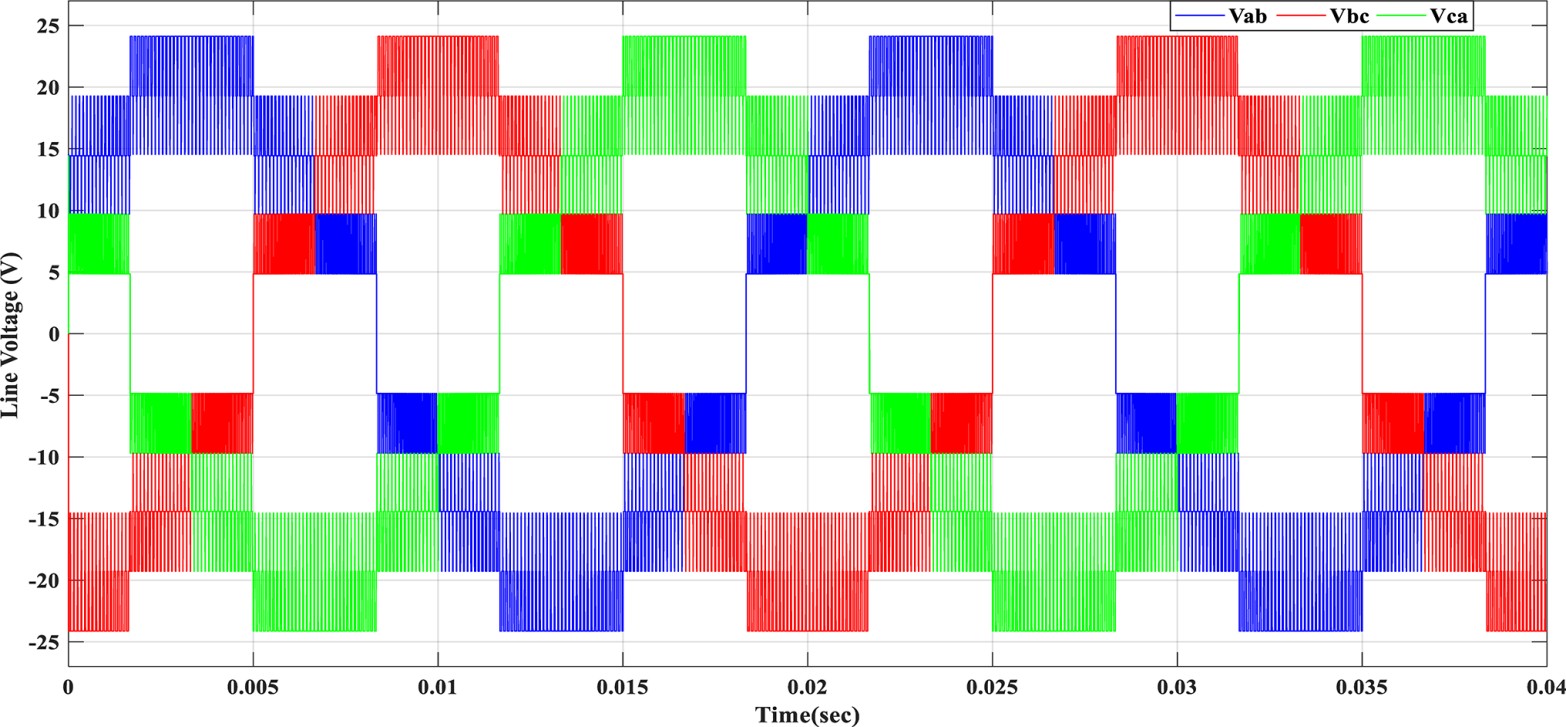
Line voltages at ${m}_{i}$= 0.95.

**Fig. 22:**
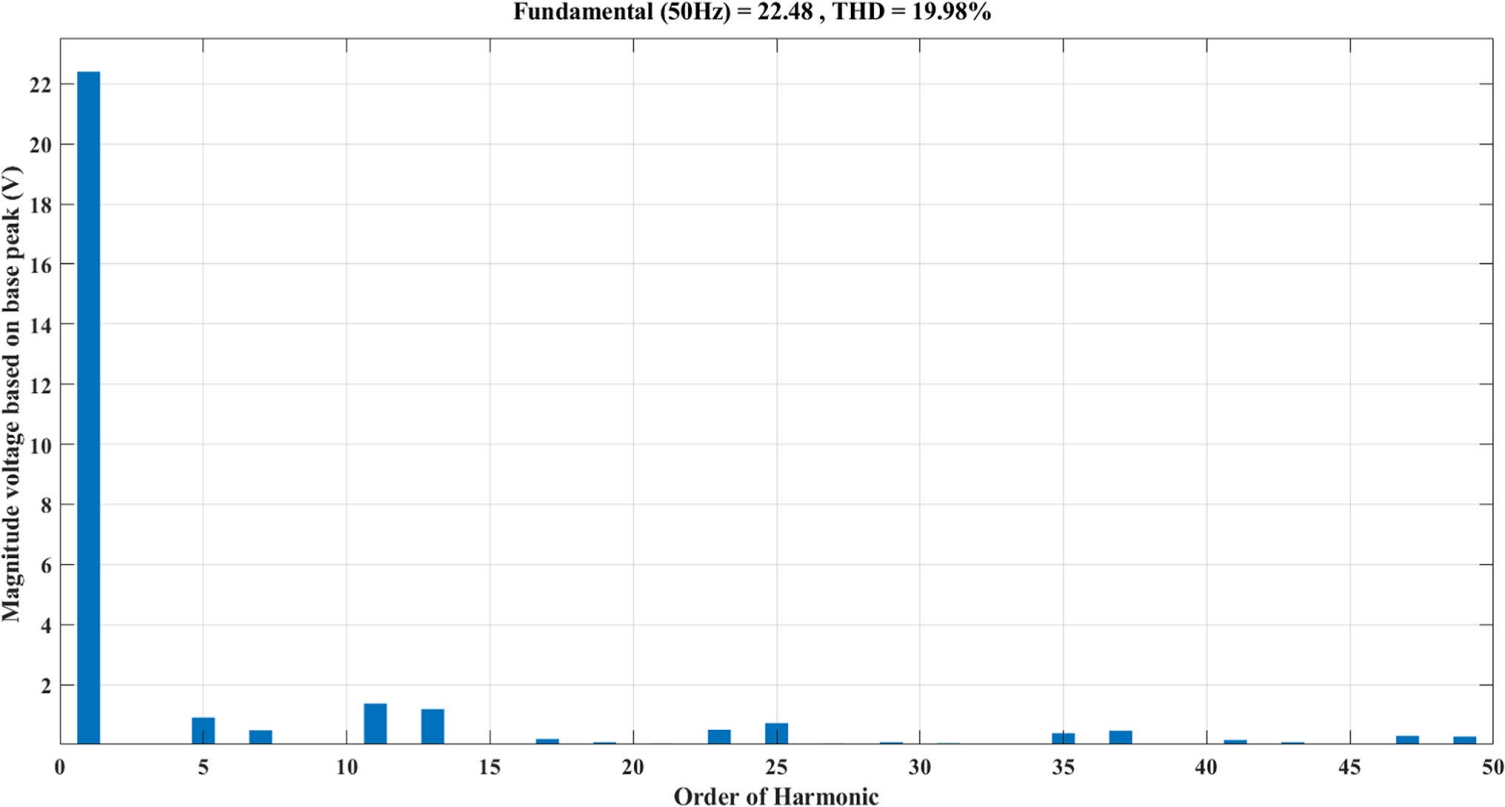
Peak magnitude spectrum for line voltage at ${m}_{i}$= 0.95.

Using the MATLAB/SIMULINK® simulation program, the inverter was tested with different values of the modulation factor (${m}_{i}$). The inverter is fed with ${V}_{1}$= 5 V and ${V}_{2}$= 10 V, ${f}_{c}$= 5 kHz. The inverter is implemented to feed a resistive-inductive load system of ${R}_{L}$ = 10 Ω, ${l}_{L}=15 m\text{H}$, and frequency ${f}_{o}$= 50 Hz. The switches are fired according to Table 6. The percent THD versus modulation factor for the SSPWM technique for the phase and line voltage is indicated in Fig. 19 and Table 7. The voltage of the three phases, line voltages, and peak magnitude spectrum for line voltage at ${m}_{i}$= 0.95 are indicated in Figs. 20, 21, and 22, respectively. The main advantage of the three-phase systems is the elimination of all triplen harmonic components in the line voltage. This makes THD of line voltage lower than THD of phase voltage. It is evident from Fig. 19 and Table 6 that the point of minimum THD manifests itself when ${m}_{i}$ is set to 0.95. Figure 20 showcases the three-phase voltages obtained, exhibiting a state of balance, whereas Fig. 20 illustrates the line voltages within the system, wherein the levels have been augmented. The FFT is employed to scrutinize the resulting output voltage, as exemplified by Fig. 22.

**Table 6:** Switching table for 7-level inverter.

V_out_		Level Generation Stage				Polarity Stage			
		S_1_	S_2_	S_3_	S_4_	Q_1_	Q_2_	Q_3_	Q_4_
0	0	0	1	0	1	1	1	0	0
V_1_	V_dc_	1	0	0	1	1	1	0	0
V_2_	2V_dc_	0	1	1	0	1	1	0	0
V_1_ + V_2_	3V_dc_	1	0	1	0	1	1	0	0

**Table 7:** Percent THD with variation of modulation factor values.

${m}_{i}$	0.1	0.2	0.3	0.4	0.5	0.6	0.7	0.8	0.9	1
$TH{D\%}_{v, ph}$	35.4	37.22	37.91	37.81	37.12	35.96	34.41	32.53	30.38	27.88
$TH{D\%}_{v, line}$	23.8	27.17	28.74	29.18	28.79	27.77	26.19	24.08	21.44	18.13

### Results implementing the suggested SPWM approach

**Fig. 23:**
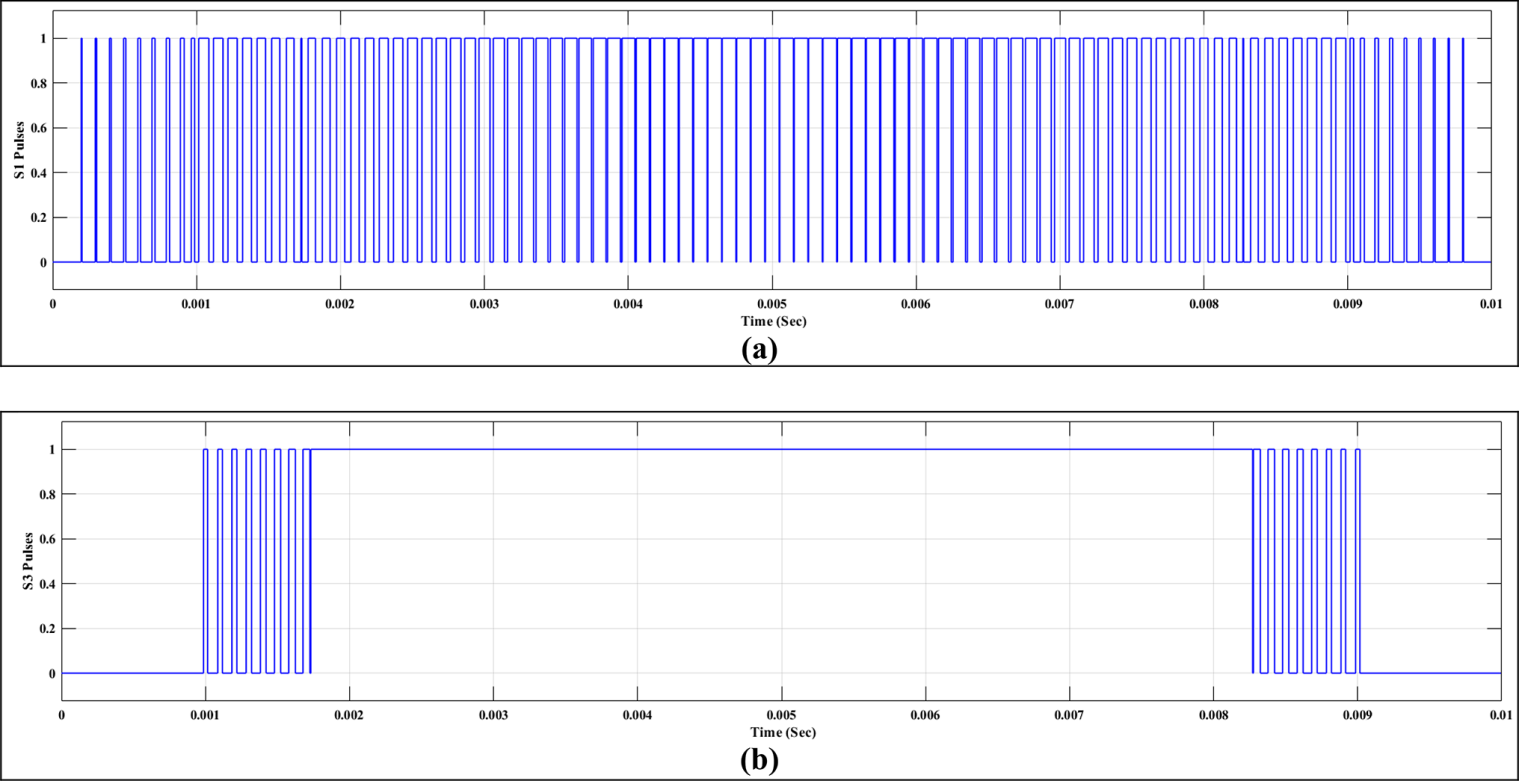
Gating pulses for the MOSFETs in Phase A.

**Fig. 24:**
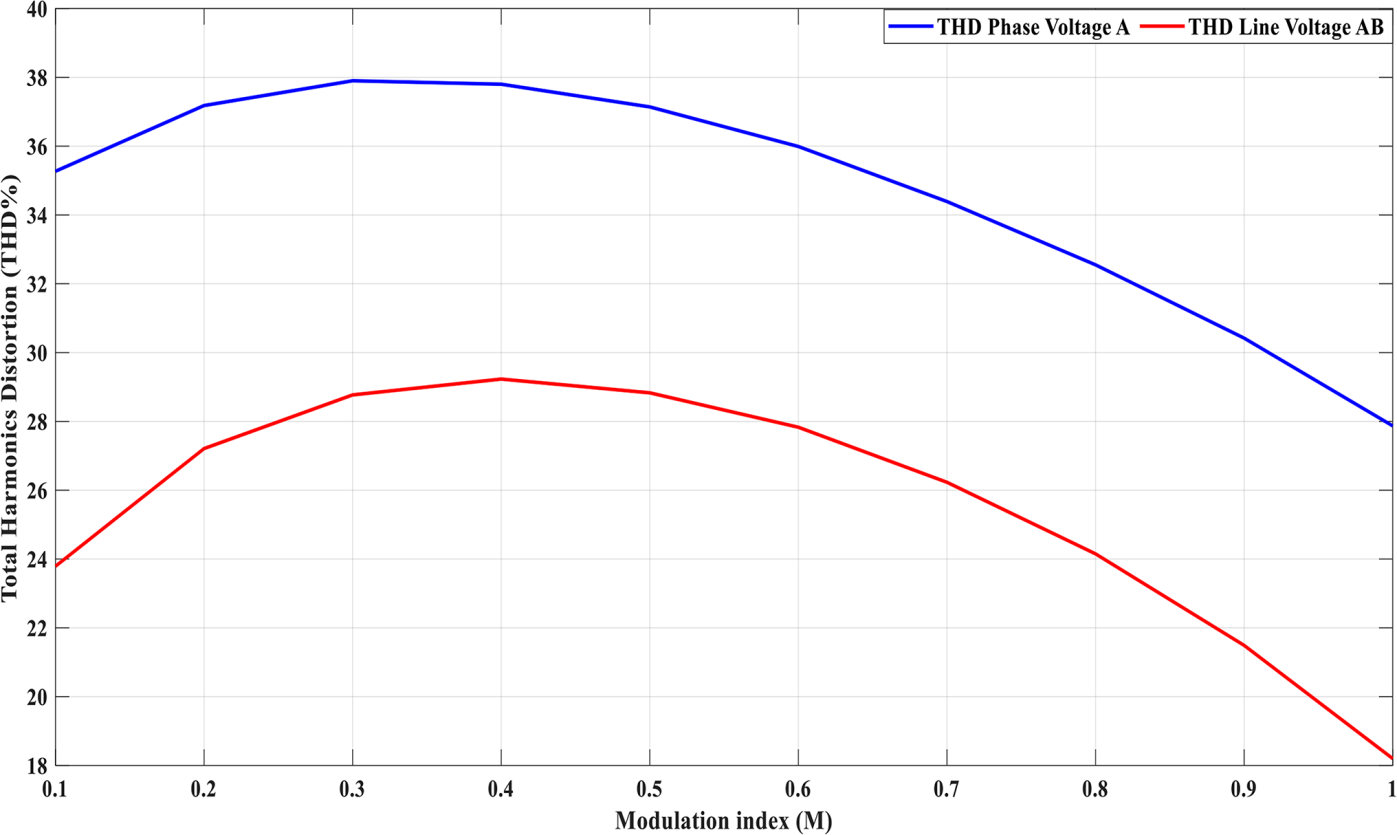
Percent THD versus modulation factor for the suggested SPWM approach.

In this case, the suggested approach discussed in “Methodology of the SSPWM technique” section is implemented using the MATLAB/SIMULINK® software for the 7-level MLI. Notably, the MLI is supplied with power through an asymmetric approach. Figure 23a and b illustrate the pulse patterns associated with switches ${S}_{1}$ and ${S}_{3}$, respectively. Furthermore, Table 8 and Fig. 24 present the percentage of the THD in both the phase and line voltages as a function of the modulation factor. Evidently, it can be observed from Fig. 24 and Table 8 that the THD percentage experiences a reduction.

**Table 8:** THD% with variation of modulation factor values.

${m}_{i}$	0.1	0.2	0.3	0.4	0.5	0.6	0.7	0.8	0.9	1
$TH{D\%}_{v, ph}$	34.27	36.18	36.9	36.8	36.14	34.99	33.39	31.55	29.42	27.87
$TH{D\%}_{v, line}$	22.79	26.21	27.77	28.23	27.83	26.83	25.23	23.15	20.49	17.2

### Results implementing the proposed ALO-SPWM control scheme

In this subsection, a case study was conducted to examine the performance of a three-phase 7-level inverter powered by the asymmetric technique. This specific type of inverter is employed to supply power to a three-phase resistive-inductive load interconnected in a star configuration. To determine the optimal solution, the parameters of the ALO algorithm, as outlined in the previous Table 3, were carefully selected through an iterative process. To obtain a solution for each modulation factor value (${m}_{i}$), the ALO algorithm is executed repeatedly, covering the entire range of ${m}_{i}$.

Table 9 presents the relationship between the modulation factor and the observed variation in switching angles within the seven-level inverter. The efforts of percent THD for both the line and phase voltages besides the load currents for the entire range of ${m}_{i}$ is indicated in the table. The THD% values for both phase voltage and line voltage were obtained through a simulation program model. Since the nature of the load arrangement is unknown, both single-phase and three-phase voltage THD% values were studied to cover different load configurations, regardless of whether they are connected in a star or delta arrangement. The proposed scheme has been thoroughly tested to assess its performance under different load conditions.

**Table 9:** Variation of THD% with ${m}_{i}$ after implementing ALO algorithm.

${m}_{i}$	Optimal switching for each voltage level			$TH{D\%}_{v, ph}$	$TH{D\%}_{v, line}$	$TH{D\%}_{i,L}$	$TH{D\%}_{i,C}$
	$Secto{r\_width}_{1}$	$Secto{r\_width}_{2}$	$Secto{r\_width}_{3}$				
0.1	0.0319	0.0611	0.1771	25.9212	18.56	0.07081	19.944
0.2	0.03981	0.0701	0.1872	25.7852	18.21	0.06611	19.326
0.3	0.0492	0.0707	0.1875	25.4911	17.69	0.06027	19.295
0.4	0.0604	0.0716	0.2519	26.2251	17.18	0.05866	20.623
0.5	0.0906	0.0951	0.3053	26.1223	16.81	0.05191	19.786
0.6	0.0762	0.1191	0.3664	25.6217	15.78	0.04976	18.928
0.7	0.1099	0.1496	0.4279	24.6931	15.63	0.04688	18.687
0.8	0.1209	0.1707	0.5013	23.4278	15.12	0.04147	18.248
0.9	0.171	0.1795	0.5648	21.7919	14.64	0.04081	10.741
1	0.151	0.2147	0.6282	19.6413	14.95	0.03879	10.028

**Fig. 25:**
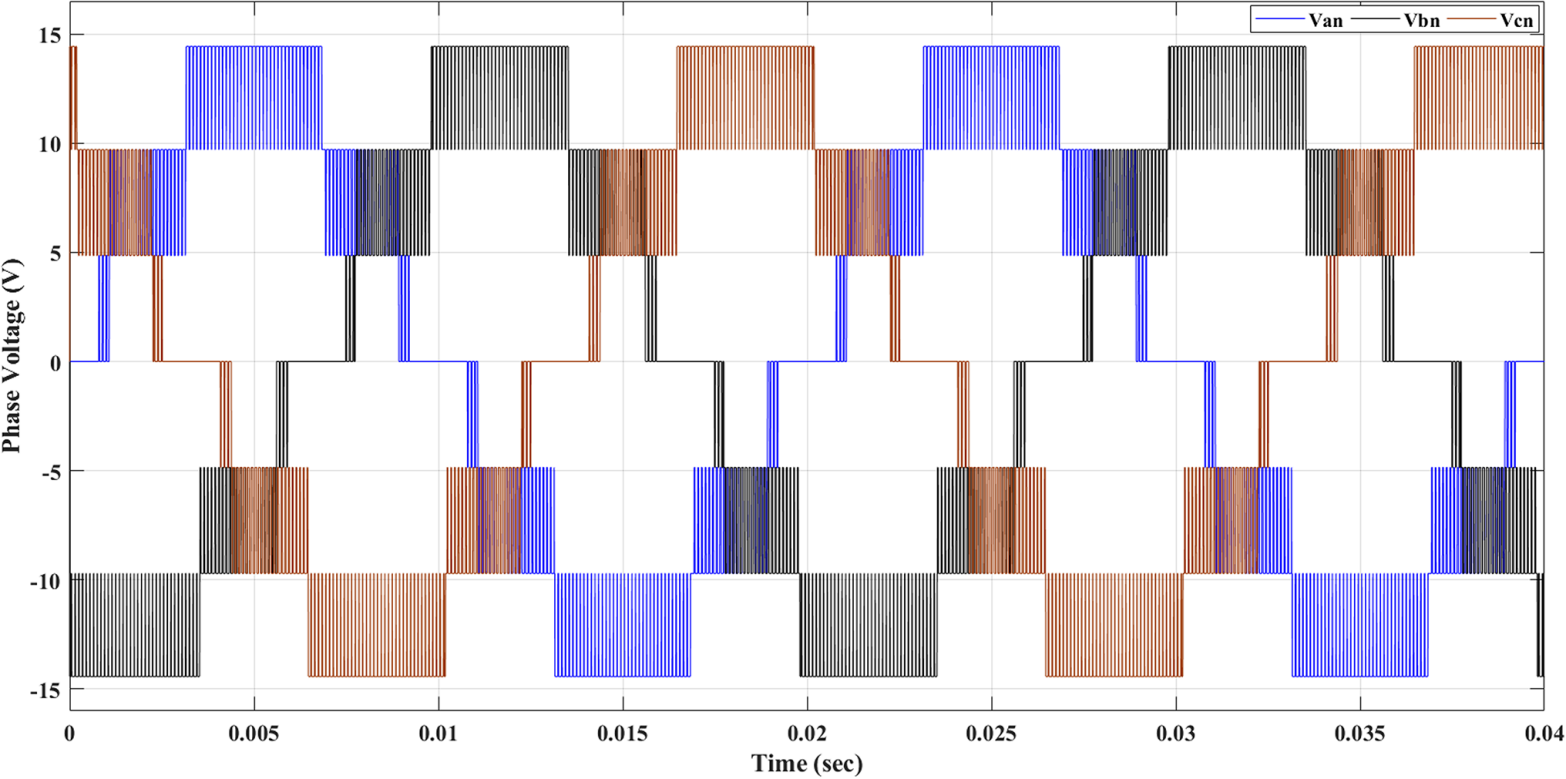
Three-Phase voltage after implementing ALO.

**Fig. 26:**
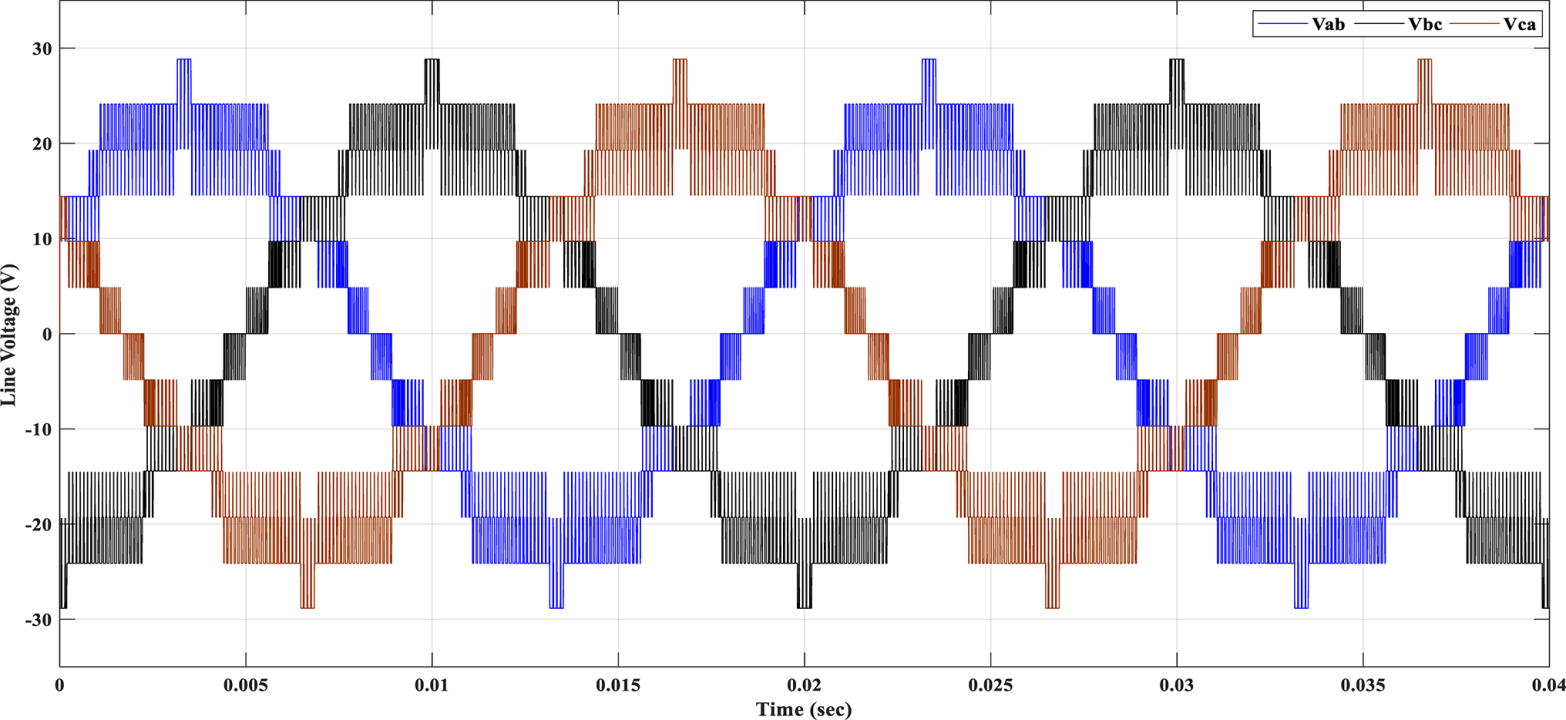
Line voltage after implementing ALO algorithm.

**Fig. 27:**
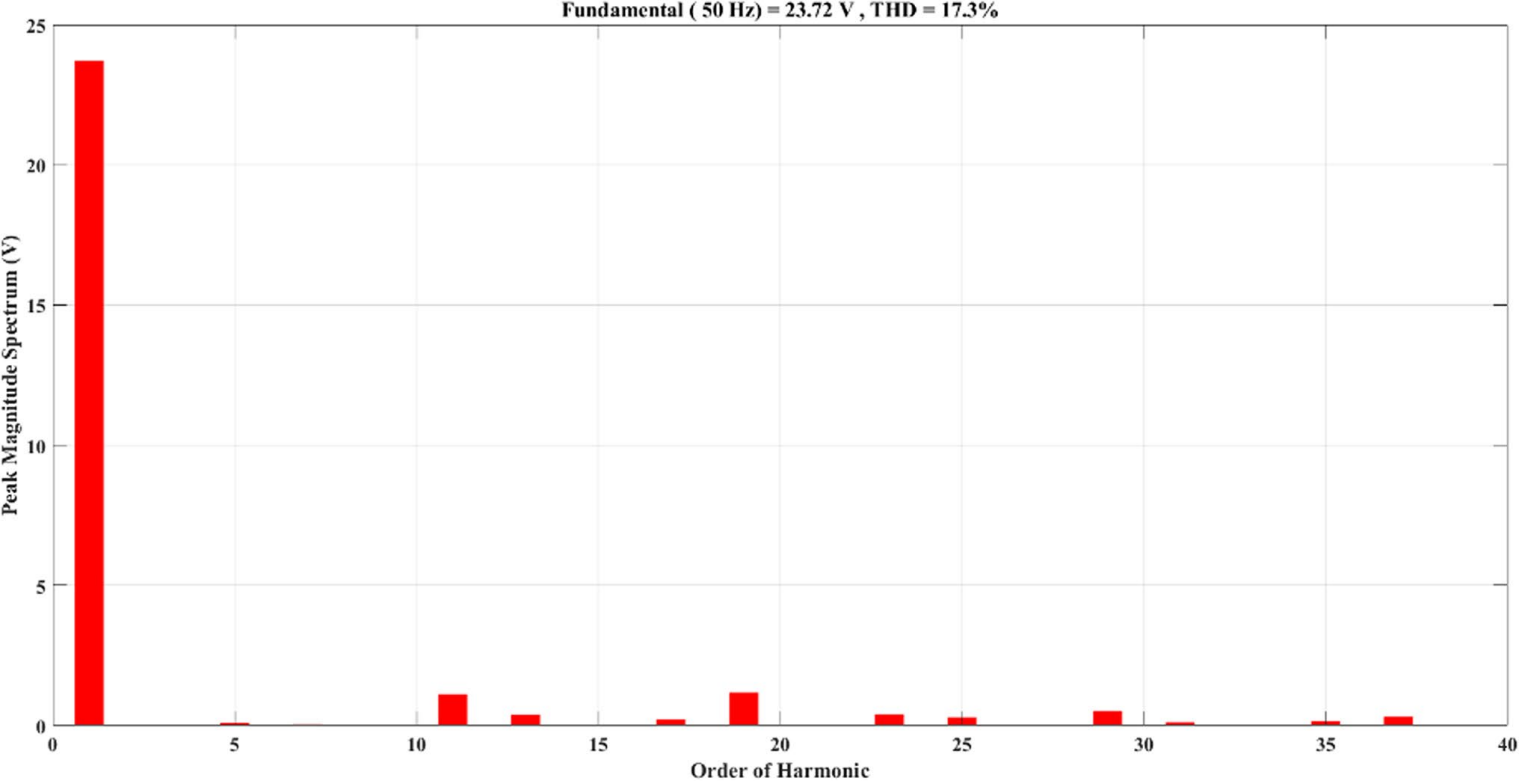
Line voltage harmonic content after implementing ALO algorithm.

The results obtained include the waveforms of the resulting voltage from the initial stage, the three-phase voltages, and the line voltages. These waveforms are depicted in Figs. 25, 26, and 27, respectively. They illustrate a well-balanced three-phase system, where the distribution of voltage among the three phases is symmetrical and equal in magnitude. This balance is crucial for promoting efficient power transmission and utilization within the system.

To evaluate its capability to maintain optimal functionality, the load configuration was changed from a RL load to a RC load, using a resistance value of ${R}_{load}=10\Omega$ and a capacitance value of ${C}_{load}= 680 \mu F$, which resulted in a leading power factor (pf) of 0.9. The results of these tests, demonstrating how the scheme responds to varying loads, are summarized in Table 9. These findings offer important insights into the scheme’s adaptability and effectiveness in providing consistent performance across various load configurations.

**Fig. 28:**
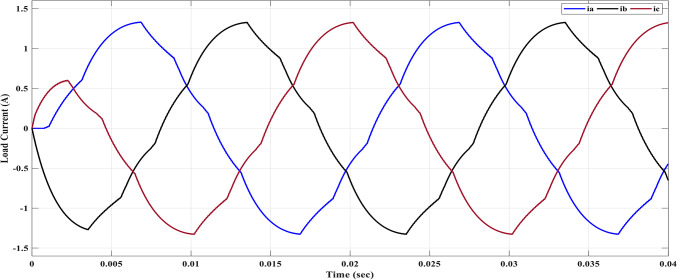
Load currents after implementing ALO algorithm for 7-level MLI.

Figure 26 shows that the line voltage exhibits an increased number of voltage levels, as indicated by Eq. (18). This leads to a waveform that closely resembles a sine wave, resulting in reduced harmonic distortions, as depicted in Fig. 27. The optimization of the switching process contributes to the improvement of the line voltage. Figure 27 provides a spectrum analysis of the line voltage, offering insights into the frequency components across all three phases. Additionally, Fig. 28 showcases the three-phase current with a resistance value of ${R}_{L}=10\Omega$ and an inductance value of ${L}_{L}=15\text{ mH}$, using the same values for ${m}_{i}$ and ${m}_{fr}$.

Compared to Fig. 22 it can be observed that the 5^th^ and 7^th^ harmonics have been eliminated because of implementing the ALO technique. ALO specifically targeted these harmonics due to their relatively larger magnitudes. By eliminating these harmonics, the objective was to achieve the optimal THD level. In Fig. 28, the load current exhibited non-sinusoidal characteristics primarily because the selected load had a relatively small inductance, and a low number of levels is 7. As a result, the waveform contained significant harmonic components. This posed a challenge in achieving the desired sinusoidal waveform. To address this issue, a filter was implemented to mitigate the harmonic content and improve the waveform quality.

The shifting between the maximum and minimum values of the load current is attributed to the nature of the load. This shifting occurs due to variations in load characteristics or load arrangement. Factors such as load type, impedance, and operating conditions can influence this shifting phenomenon, leading to fluctuations in the load current between its maximum and minimum values.

### Comparison with the approaches used in the literature

Table 10 provides a comprehensive comparison between the implementation of the ALO, GA, PSO, suggested SPWM approach, and the SSPWM technique implemented in the literature. These approaches are implemented on the adopted 7-level MLI. Specifically, the comparison focuses on THD for phase voltage, line voltage, and load currents with modulation factor variation. The purpose of this comparison is to ensure and verify the effectiveness of the ALO technique concerning the adopted MLI and its application in obtaining an optimal switching angle.

**Table 10:** Efforts of the proposed ALO-SPWM control scheme in comparison to the GA and PSO techniques.

Approach	${m}_{i}$	Optimal switching for each voltage level			$TH{D\%}_{v, ph}$	$TH{D\%}_{v, line}$	$TH{D\%}_{i,L}$
		$Secto{r\_width}_{1}$	$Secto{r\_width}_{2}$	$Secto{r\_width}_{3}$			
SPWM (Equally Stepped)	0.1	0	0.333333	0.666667	34.27	22.79	0.07081
	0.2				36.18	26.21	0.0661
	0.3				36.9	27.77	0.06027
	0.4				36.8	28.23	0.05866
	0.5				36.14	27.83	0.05191
	0.6				34.99	26.83	0.04976
	0.7				33.39	25.23	0.04688
	0.8				31.55	23.15	0.04147
	0.9				29.42	20.49	0.04081
	1				27.87	17.2	0.03879
GA	0.1	0.02647912	0.1479641	0.36542176	26.9237812	23.19	0.041876
	0.2	0.0578143	0.1475216	0.4127458	26.4698727	21.78	0.03257
	0.3	0.0415897	0.2417311	0.3147521	27.1458771	20.98	0.04971
	0.4	0.0326811	0.1254896	0.3125864	28.8147833	20.35	0.05987
	0.5	0.0566428	0.1030515	0.3106559	28.5261345	19.26	0.05191
	0.6	0.0815771	0.1432571	0.3595431	26.4465811	18.89	0.04976
	0.7	0.03720678	0.1559617	0.4328029	25.5261345	17.88	0.04688
	0.8	0.0042485	0.0952930	0.3757247	22.5332242	17.68	0.04147
	0.9	0.0465555	0.1506749	0.5211094	21.4208030	16.11	0.04081
	1	0.0467005	0.1725954	0.5926912	20.2469405	**16.84**	0.03879
ALO	0.1	0.0319	0.0611	0.1771	24.921278	18.56	0.07081
	0.2	0.03981	0.0701	0.1872	24.785221	18.21	0.06611
	0.3	0.0492	0.0707	0.1875	24.491173	17.69	0.06027
	0.4	0.0604	0.0716	0.2519	25.225156	17.18	0.05866
	0.5	0.0906	0.0951	0.3053	25.122327	16.81	0.05191
	0.6	0.0762	0.1191	0.3664	24.621796	15.78	0.04976
	0.7	0.1099	0.1496	0.4279	23.693134	15.63	0.04688
	0.8	0.1209	0.1707	0.5013	22.427878	15.12	0.04147
	0.9	0.171	0.1795	0.5648	20.791901	14.64	0.04081
	1	0.151	0.2147	0.6282	18.641310	**14.95**	0.03879
PSO	0.1	0.00185	0.08461	0.22541	26.852168	24.214	0.181226
	0.2	0.01287	0.09562	0.28622	26.625831	22.651	0.167103
	0.3	0.04767	0.08977	0.34765	27.294734	23.724	0.199561
	0.4	0.07146	0.09861	0.28892	26.521056	23.451	0.159878
	0.5	0.08816	0.11951	0.35632	25.034716	21.725	0.101823
	0.6	0.08654	0.1201	0.32754	25.16287	21.205	0.089864
	0.7	0.17682	0.13981	0.43872	24.781278	20.618	0.095821
	0.8	0.12582	0.13267	0.52781	23.265178	19.752	0.084262
	0.9	0.11691	0.18195	0.57982	22.670127	18.256	0.078214
	1	0.13698	0.28527	0.68721	21.978121	**17.144**	0.060827

Upon analyzing the data presented in Table 10, it becomes evident that the implementation of the ALO technique results in a reduced THD for line voltage, achieving 11% compared with the GA technique, and 12.8% compared with the PSO, which yields a THD of 14.95%. Furthermore, the assessment of Fig. 30, which depicts the THD% of phase voltage, respectively, against the modulation factor, provides further insights. These Figures demonstrate that the proposed SPWM control scheme, optimized using the ALO algorithm (ALO-SPWM), outperforms the results obtained through the SPWM control scheme tuned using the GA technique (GA-SPWM) and the PSO (PSO-SPWM) technique. The suggested scheme exhibits a significant reduction in THD values for both phase and line voltages. Moreover, the ALO algorithm offers the advantage of requiring fewer parameters compared to the GA algorithm, rendering it an attractive choice for optimization tasks.

**Fig. 29:**
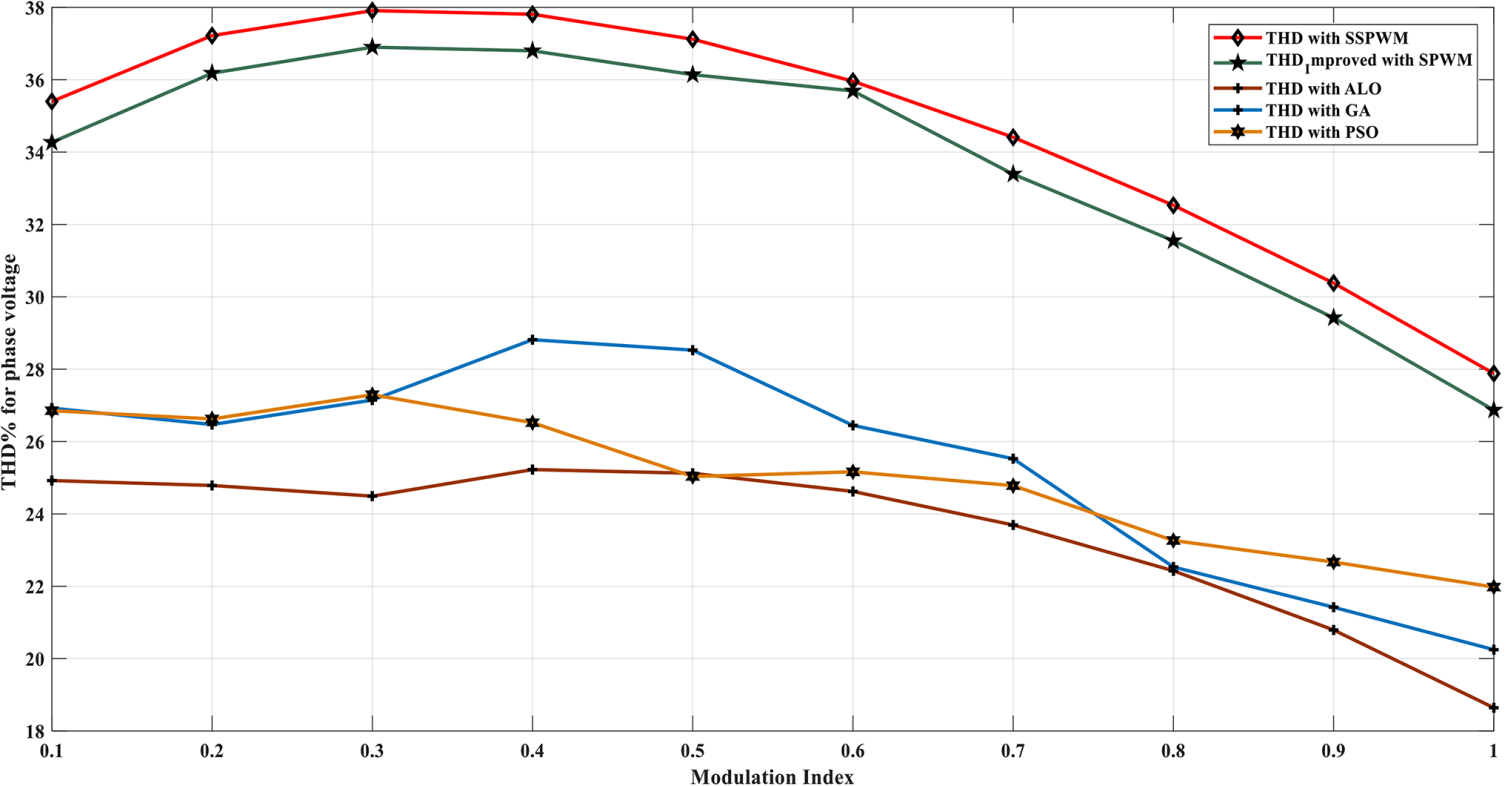
THD% of the phase voltage vs. the modulation factor.

In conclusion, the comparison presented in Table 10, along with Fig. 29 unequivocally supports the superiority of the proposed ALO-SPWM over the GA-SPWM and the PSO-SPWM. The ALO algorithm demonstrates its capability to effectively reduce THD values and showcases its potential as a more streamlined and efficient optimization technique. To meet the requirements stated in the IEEE standard, it is necessary to reduce the THD to achieve a level of 8% as stated in reference^56^. To accomplish this, an approach must be employed to minimize the presence of harmonics. In this case, an LC passive filter has been chosen and designed to effectively achieve this objective in the following section.

The results show that the suggested approaches of the improved SPWM, GA-SPWM, and ALO-SPWM have successfully reduced the THD compared to the traditional SSPWM technique. The THD reduction percentages are 5.1% for improved SPWM, 6.9% for GA-SPWM, 5.5% for PSO-SPWM, and 17.5% for ALO-SPWM when compared to SSPWM. Although the THD has significantly decreased and the need for a filter is reduced resulting in cost savings, the THD values obtained with the applied techniques still do not meet the IEEE standard, thus further improvements will be implemented to achieve the desired level of harmonic distortion. The IEEE standard of 8% for THD is still not satisfied. Therefore, additional measures are required to reduce the THD further and meet the standard. In the next subsection, a small filter has been designed to address this issue.

### Implementation of the LC filter

Passive filters, renowned for their uncomplicated design and economical nature, stand as the prevailing and cost-effective remedy employed to mitigate the predicaments brought about by harmonic distortion. Within the confines of this scholarly document, an exquisite lossless LC filter is meticulously devised, wherein substitution of the following parameters into Eq. (27), elegantly disclosed in “Harmonic mitigation filters” section is undertaken: ${f}_{1}$= 50 Hz, ${V}_{ph}$= 35 V and ${P}_{Load}$ = 25 W.

**Fig. 30:**
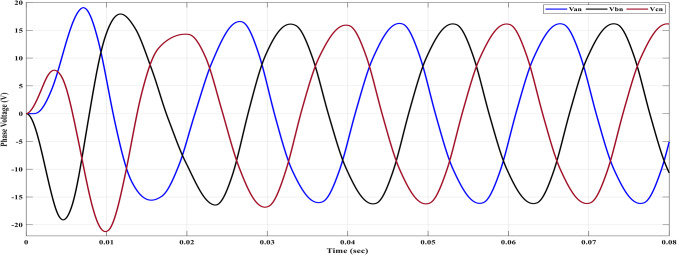
Three-phase voltages after filter side.

**Fig. 31:**
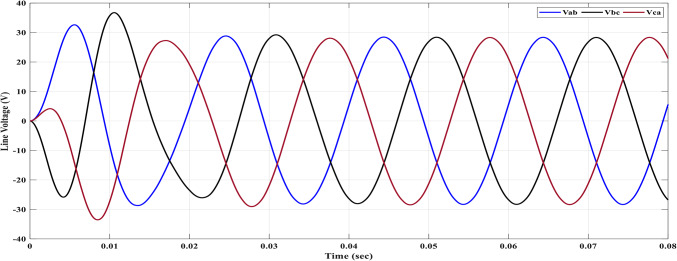
Line voltages after filter side.

**Fig. 32:**
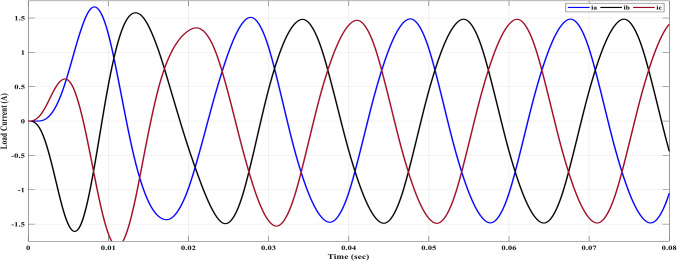
Load currents after filter side.

The parameters essential for the design are acquired through an intricate process as delineated: The maximum capacitance (${C}_{max}$) is determined to be 450 μF, with an additional selection of ${C}_{f}$ at a value of 330 μF. By skillfully solving Eq. (28) at the dominant harmonic, the maximum inductance (${L}_{max}$) is computed to possess a magnitude of 15 mH. Furthermore, the filter inductance (${L}_{f}$) is deliberately chosen to be 10 mH. Upon subjecting the selected filter to the maximum power delivery scenario, it is revealed that the THD of the phase voltage stands at approximately 3.8%. Additionally, the THD for the line voltage amounts to approximately 0.13%. Figures 30 and 31 showcase the outcome of the load phase voltage and line voltage, respectively. These Figures depict a harmoniously balanced three-phase system, characterized by pristine sinusoidal waveforms. The resultant load current is vividly depicted in Fig. 32.

The peak values observed in the first cycle of Figs. 30, 31, and 32 can be attributed to transient conditions resulting from the combined effects of the load, filter, and inverter. When the load, filter, and inverter are operating together, these spikes occur due to the dynamic response during the initial transient period. These transient spikes are a normal occurrence and are expected as the system stabilizes and settles into a steady-state operation.

The suggested approach has been thoroughly evaluated, with the carrier signal frequency varied from 5 kHz to higher frequencies. Detailed analysis reveals that this frequency modulation does not significantly affect the (THD) value. This result highlights the scheme’s robustness and stability, demonstrating its capacity to deliver consistent performance despite changes in carrier signal frequency. Consequently, this reinforces the proposed scheme’s viability and reliability for its intended use.

## Experimental validation

**Fig. 33:**
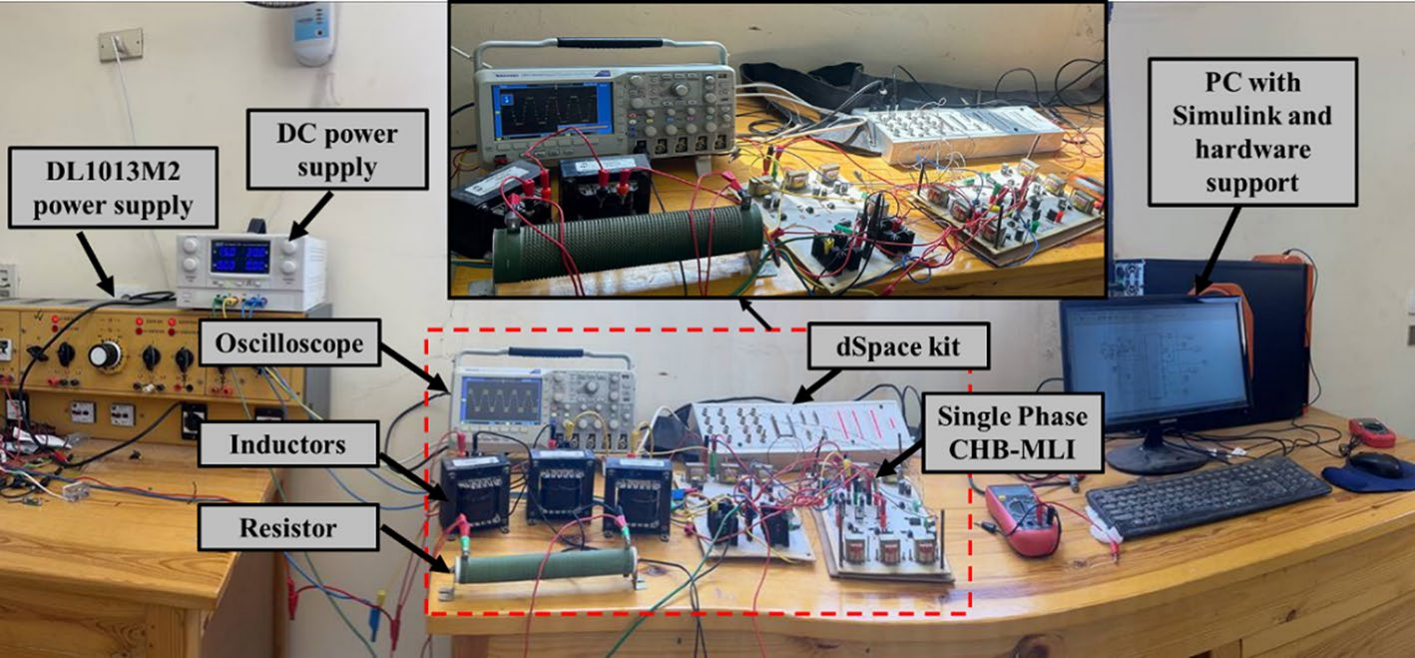
Experimental validation setup for a single-phase 7-level inverter.

A functional prototype of a single-phase 7-level inverter has been successfully developed. This prototype incorporates 500 V, 20 A power MOSFETs (IRFP460) as the primary switching devices, providing reliable and efficient operation. The 7-level structure is achieved with only eight switches, showcasing a streamlined design approach. The gate signals necessary for precise switching are derived from the dSPACE R&D controller board through a digital optocoupler interface circuit (4N38). The prototype was implemented using MATLAB/Simulink® software, enabling effective design, simulation, and optimization. Measurements are conducted using a digital oscilloscope (MCP lab electronics), ensuring accurate performance evaluation. The experimental setup illustrated in Fig. 33 serves as a valuable reference for generating test findings, contributing to a comprehensive understanding of the prototype’s capabilities.

To validate the operational efficacy of the implemented seven-level inverter, rigorous testing was conducted using DC sources. The selected DC sources, denoted as ${v}_{1}=15 \text{V}$ and ${v}_{2}=30 \text{V}$, served as the input voltages for the inverter system. Through meticulous experimentation and analysis, it was observed that the resulting ${V}_{rms}$ value of the resulting voltage attained an approximate magnitude of 35 V. To achieve a power factor of 0.9 lag, the seven-level inverter was subjected to a load consisting of a resistance ${R}_{L}=10\Omega$ and an inductance ${L}_{L}=15\text{ mH}$. This RL load configuration was carefully chosen to ensure the desired power factor, these test results confirm the successful operation of the seven-level inverter, exhibiting its ability to efficiently convert the supplied DC voltages into a stable and controlled AC resulting voltage at the desired level.

**Fig. 34:**
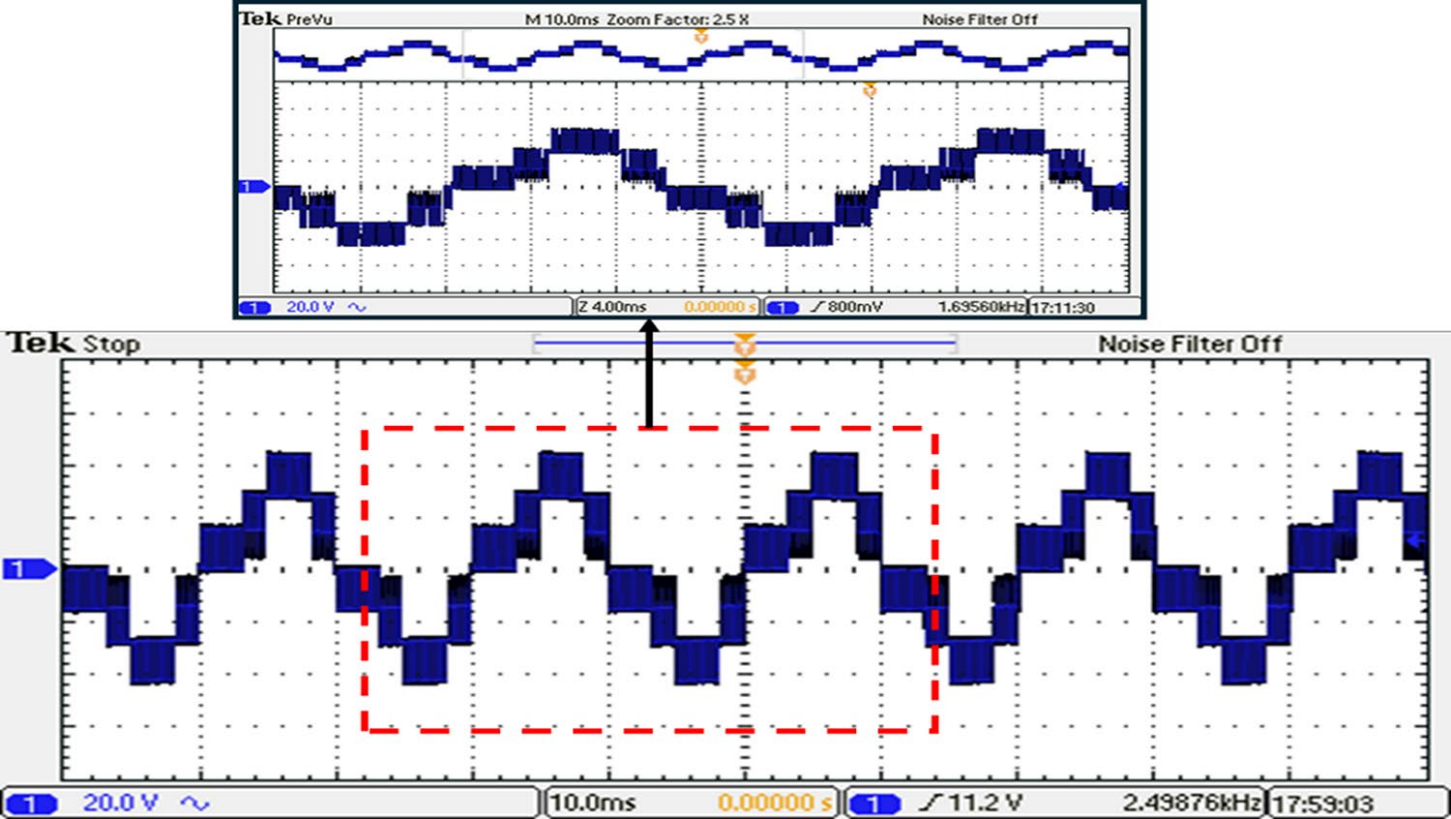
Resulting voltage at ${m}_{i}=1$ and ${m}_{{f}_{r}}=100$ before optimization technique.

**Fig. 35:**
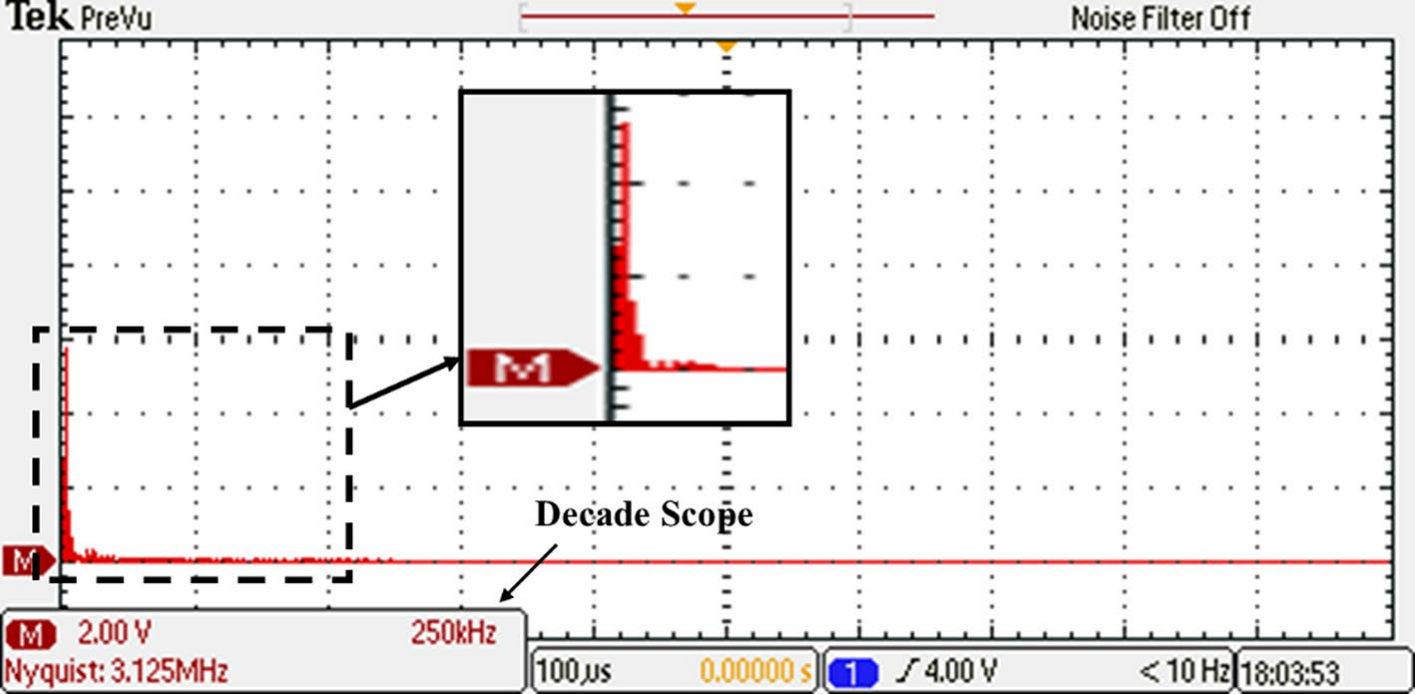
Resultant spectrum at ${m}_{i}=1$ and ${m}_{{f}_{r}}=100$ before optimization technique.

**Fig. 36:**
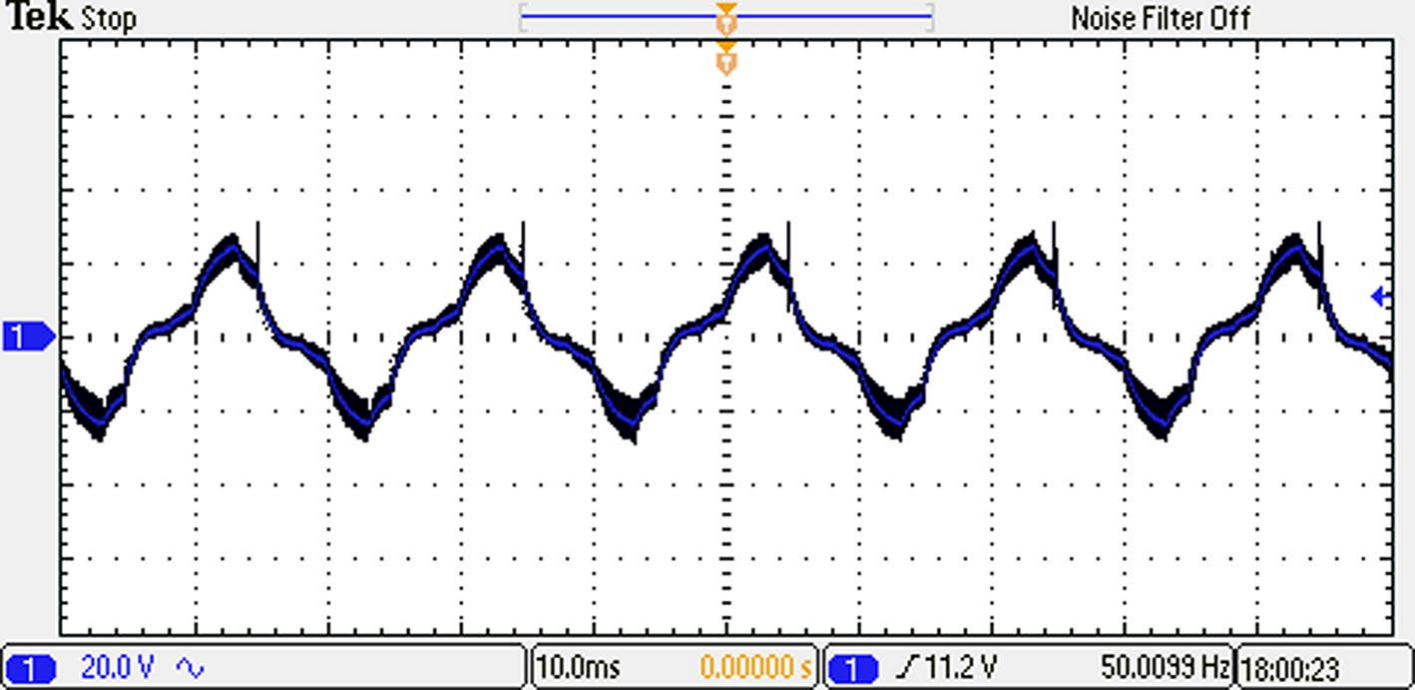
Resulting current with RL load at ${m}_{i}=1$ and ${m}_{{f}_{r}}=100$ before optimization technique.

Before undergoing optimization, the performance of the inverter was evaluated using the suggested SPWM approach with an equally stepped method. The resulting voltage waveform obtained from this initial testing phase is illustrated in Fig. 34, providing a visual representation of the voltage signal characteristics. To gain further insights into the frequency components present in the resulting output voltage, an FFT analysis was performed, and the corresponding spectrum is displayed in Fig. 35. This spectrum analysis aids in understanding the harmonic content and frequency distribution within the resulting output voltage signal. Additionally, Fig. 36 demonstrates the load current waveform at a modulation factor of 1, showing the behaviour and shape of the current flowing through the load during this preliminary testing phase. These waveform evaluations provide valuable information regarding the efficiency and effectiveness of the inverter under the given modulation conditions, serving as a basis for subsequent optimization efforts.

Various factors can give rise to notches or distortions in the output current waveform depicted in Fig. 36. These factors encompass voltage imbalance issues stemming from disparities among voltage levels. Notches and distortions may also be influenced by switching frequencies, particularly under specific load conditions. The introduction of dead time, employed to prevent the simultaneous conduction of power devices, can further contribute to waveform irregularities. Moreover, the behaviour of the MLI can be influenced by nonlinear load impedance, such as certain power electronics or motor drives, thereby resulting in waveform variations.

**Fig. 37:**
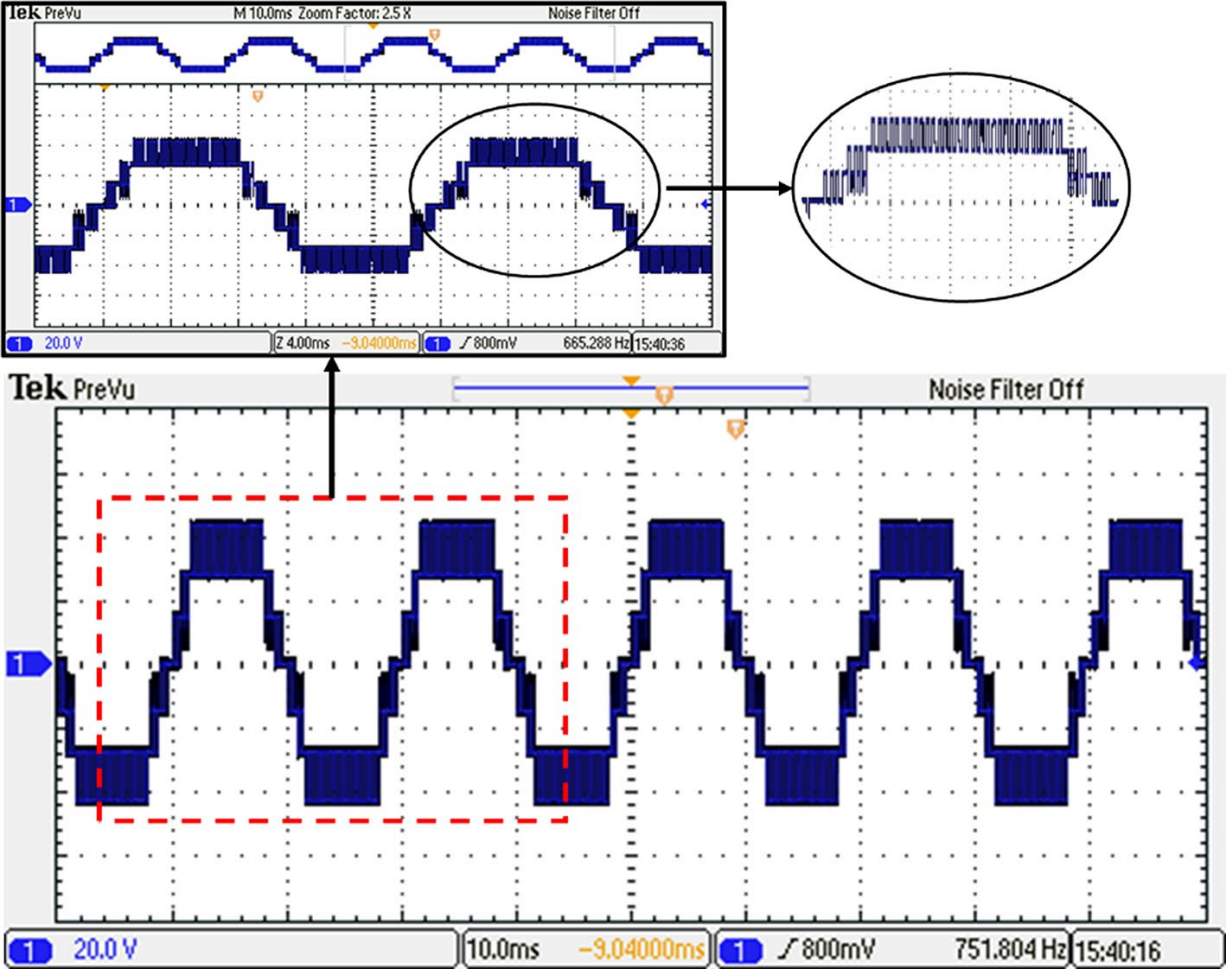
Resulting voltage at ${m}_{i}=1$ and ${m}_{{f}_{r}}=100$ after implementing ALO technique.

**Fig. 38:**
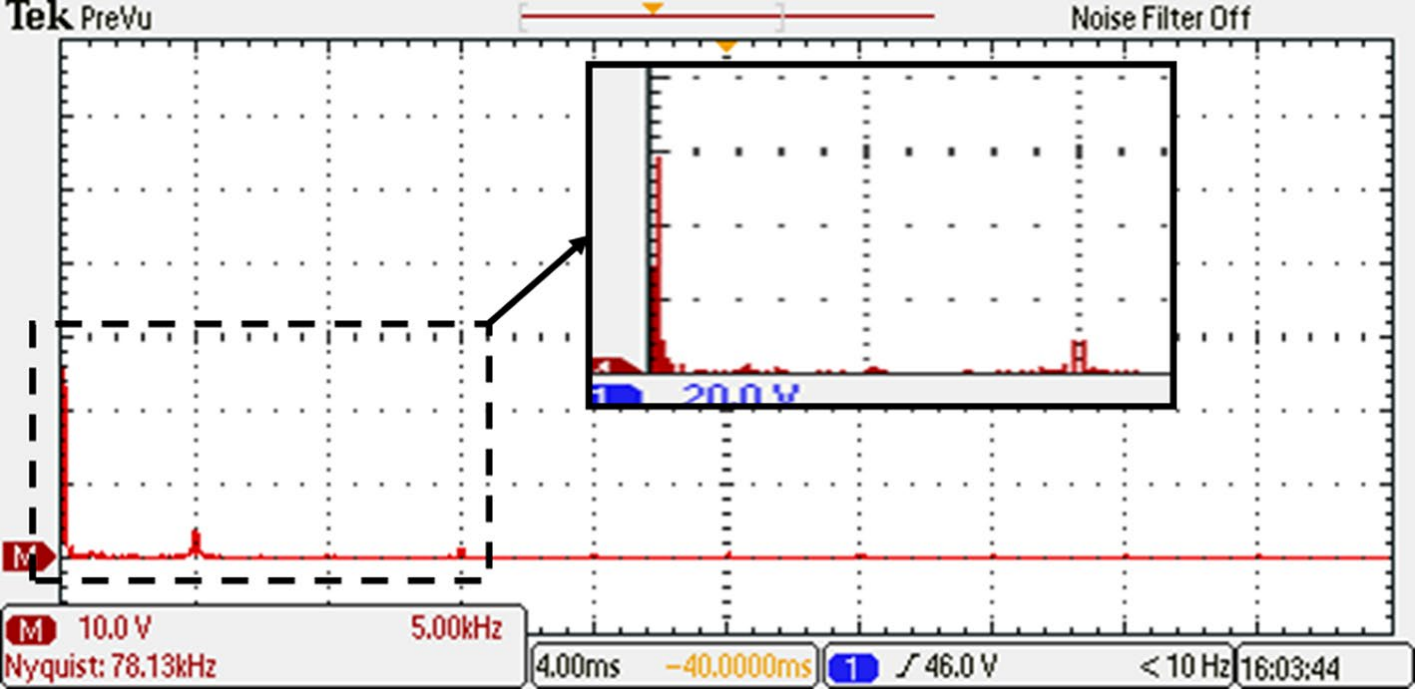
Resultant spectrum at ${m}_{i}=1$ and ${m}_{{f}_{r}}=100$ after implementing ALO technique.

The inverter underwent rigorous testing to assess its performance. The testing phase was conducted to evaluate the effectiveness of the ALO optimization in improving the inverter’s operation. The resulting voltage waveform of the seven-level from the optimized inverter under examination is visually depicted in Fig. 37, showcasing the temporal characteristics and shape of the generated voltage signal. Furthermore, Fig. 38 presents the spectrum analysis of the resulting voltage, providing valuable insights into the frequency components present in the signal at the specified load and modulation factor.

**Fig. 39:**
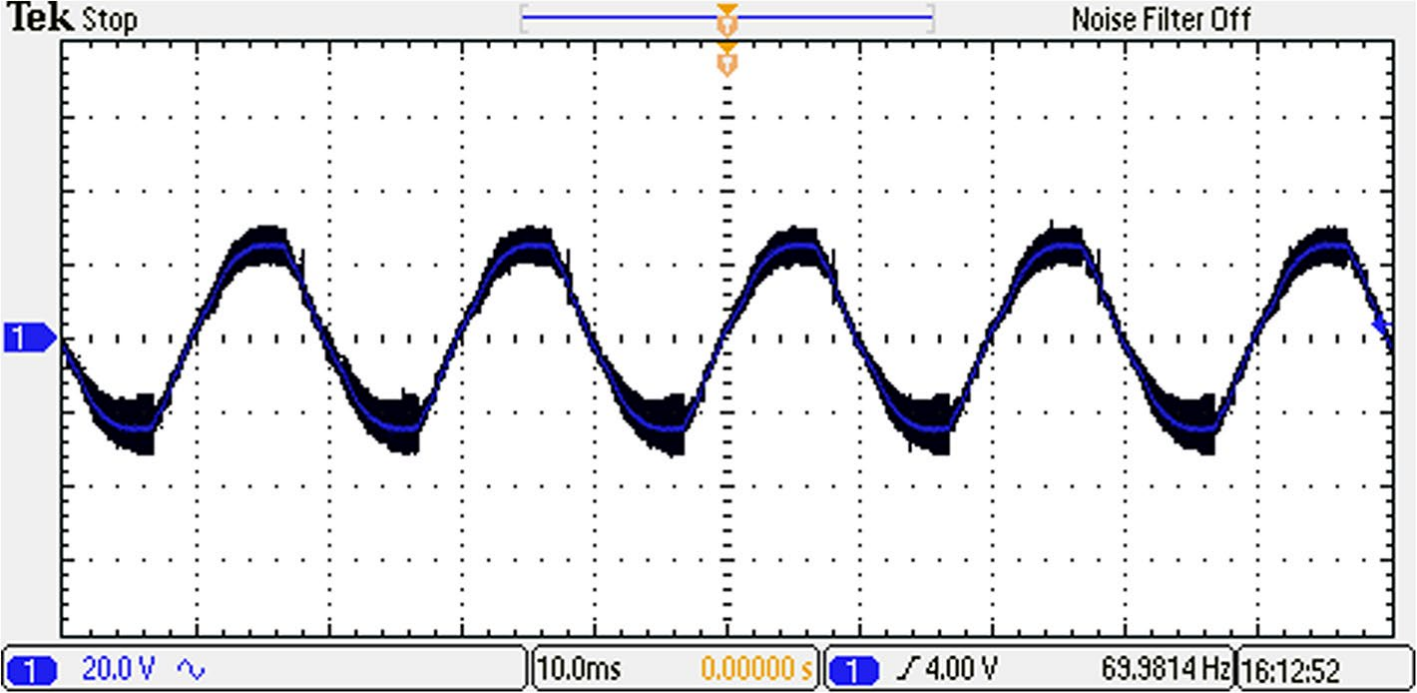
Resulting current with RL load at ${m}_{i}=1$ and ${m}_{{f}_{r}}=100$ after implementing ALO technique.

The load current waveform is illustrated in Fig. 39, revealing the behaviour and shape of the current flowing through the load. Notably, there is a discernible improvement in the sinusoidal nature of the load current, indicating a reduction in harmonics and a closer resemblance to a pure sinusoidal waveform. This enhancement in the quality of the current-waveform can be attributed to the operation of the seven-level inverter, which effectively mitigates distortions and promotes a more desirable sinusoidal current flow through the load.

**Fig. 40:**
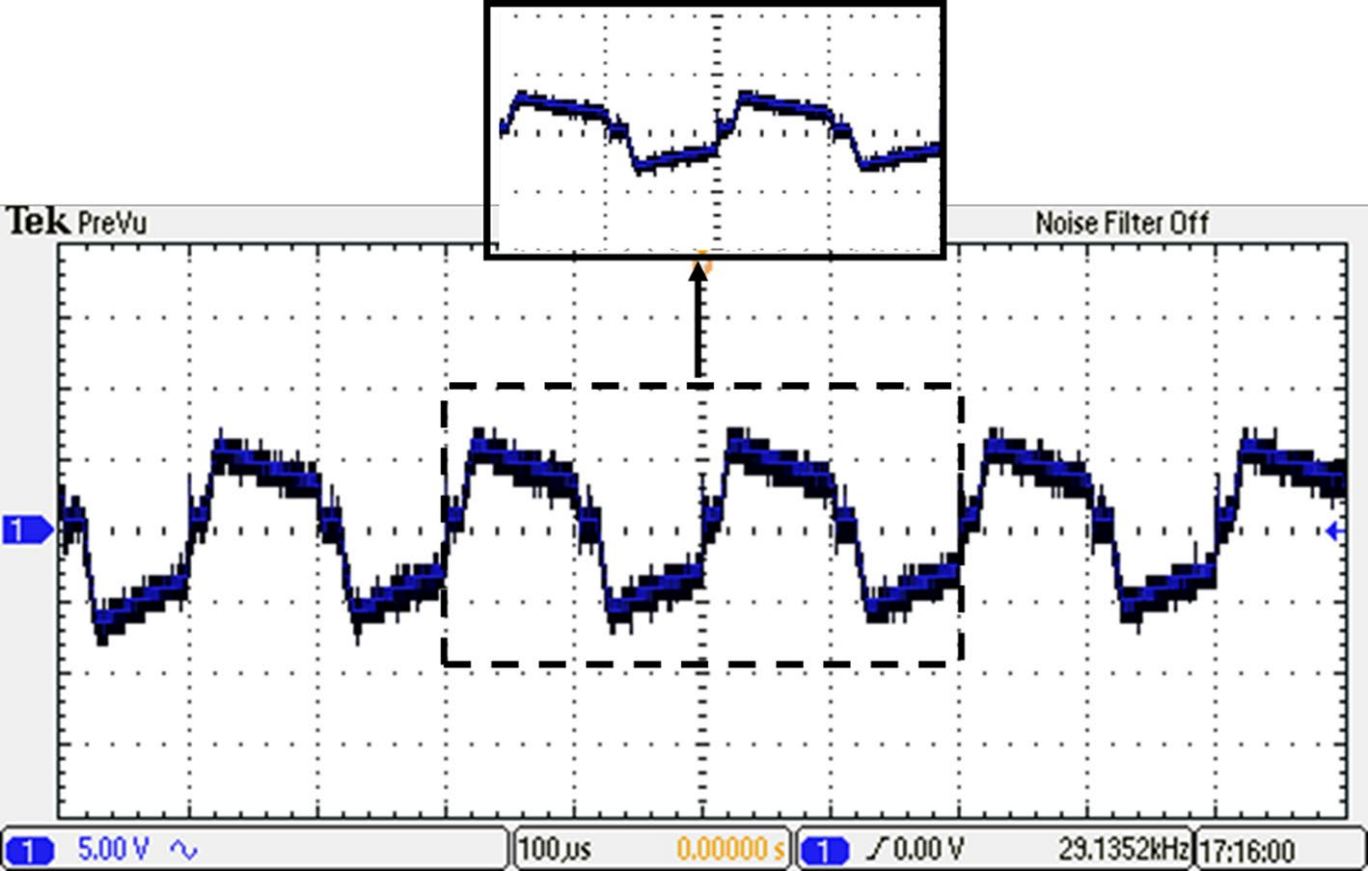
Resulting current with RC load at ${m}_{i}=1$ and ${m}_{{f}_{r}}=100$ after implementing ALO technique.

To evaluate the effectiveness of the proposed scheme, we transitioned from an inductive load to a capacitive load with a leading power factor of 0.9. This change was carried out to analyze the system’s performance across various load conditions. The load current for the capacitive configuration, with $R_{load} = 10 \;\Omega$, a capacitance per phase of $C_{load} = 680\;\mu {\text{F}}$ per phase, is depicted in Fig. 40.

The experimental results for a single-phase seven-level inverter validated the simulation results obtained using the suggested SPWM method with the equally stepped method and based on the ALO technique which is described in the “Simulation results and discussion” section. Experimental results showed a good match and similarity with the simulation results.

## Conclusion

In this paper, an innovative asymmetric MLI topology has been implemented successfully that surpasses conventional MLIs in terms of both performance and efficiency. By adopting this topology, a remarkable increase in the number of specific levels while minimizing the number of switching components required has been achieved. To ensure optimal operation of the CHB-MLI, a novel control scheme was introduced in this paper. The approach utilizes a single modulating signal, a single carrier, and a single triangle signal, all operating at the same modulating frequency. This approach significantly simplifies the switching patterns and reduces the overall complexity of the implemented MLI. To validate the effectiveness of the suggested approach, a comprehensive comparison has been introduced with the widely used SSPWM technique found in existing literature. Notably, our suggested approach exhibits a significantly lower THD compared to SSPWM. This reduction in THD not only enhances the overall quality of the resulting waveform but also allows for a considerable reduction in filter size and cost. Furthermore, the code-based approach ensures simplicity and cost-effectiveness in implementation, utilizing a low-cost DSP. A meticulously analyzed output of the three-phase inverter has been implemented, comparing it with both symmetric and asymmetric DC sources. Through optimization techniques, specifically the application of the ALO algorithm, GA algorithm, and PSO algorithm, THD is successfully reduced from 27.88% to an impressive 19% for the asymmetric type. Simulation results showed that the suggested approaches of the improved SPWM, GA-SPWM, and ALO-SPWM have successfully reduced the THD compared to the traditional SSPWM technique. The THD reduction percentages are 5.1% for improved SPWM, 6.9% for GA-SPWM, and 17.5% for ALO-SPWM when compared to SSPWM. To meet the stringent standards set by the IEEE, an LC filter is designed with minimal size and requirements, effectively reducing costs without compromising performance. Experimental validation of the suggested scheme was conducted using a dSPACE R&D controller board, which unequivocally confirmed the robustness and effectiveness of the proposed approach. Through this study, this paper not only introduced a superior asymmetric MLI topology but also demonstrated its exceptional performance through comprehensive analysis and experimentation. The findings hold immense promise for advancing the field of power systems and have significant implications for the design and implementation of efficient and cost-effective inverter systems.

## Data Availability

The data that support the findings of this study are available from the corresponding author upon reasonable request.
